# LINC01518 functions as an oncogene in head and neck squamous cell carcinoma (HNSCC) by modulating *miR-1-3p*/Slug and *miR-216b-5p*/GRP78 axis

**DOI:** 10.1038/s41598-025-06934-6

**Published:** 2025-07-02

**Authors:** Swati, Shraddha Tripathi, Bakhya Shree, Suryansh Sengar, Amit Mishra, Vivek Sharma

**Affiliations:** 1 Department of Biological Sciences, Birla Institute of Technology and Science, Pilani, Hyderabad Campus, Jawahar Nagar, Kapra Mandal, Medchal District, Telangana 500078 India; 2 Cellular and Molecular Neurobiology Unit, Indian Institute of Technology Jodhpur, Jodhpur, Rajasthan India

**Keywords:** HNSCC, TGF-β, LINC01518, ceRNA, *miR-1-3p*, *miR-216b-5p*

## Abstract

HNSCC is a highly aggressive cancer of the head and neck region, and there is an urgent need to find novel potential targets for its diagnosis and treatment. Long non-coding RNAs (lncRNAs) have emerged as important therapeutic and diagnostic targets for multiple cancers, including HNSCC. LINC01518 promotes the proliferation of oesophageal cancer cells, but the involvement of LINC01518 in HNSCC pathophysiology is unknown. We show that LINC01518 expression is significantly upregulated in high-grade HNSCC tumor samples in comparison to normal tissue, and transforming growth factor- β (TGF-β) promotes LINC01518 expression in HNSCC cell lines. Loss-of-function studies suggest that LINC01518 promotes cell proliferation, migration, and invasion in HNSCC cells. In addition, LINC01518 depletion sensitizes HNSCC cells to cisplatin-mediated apoptosis. Mechanistically, LINC01518 acts as a competitive endogenous RNA and binds to *miR-1-3p* and *miR-216b-5p*, resulting in up-regulation of their target genes Slug and GRP78, respectively. Our findings suggest that LINC01518 is an attractive therapeutic target for HNSCC.

**Supplementary Information:**

The online version contains supplementary material available at 10.1038/s41598-025-06934-6.

## Introduction

HNSCC is the seventh most common cancer worldwide; the recent GLOBOCAN data (2022) estimated 946,456 new cases and 482,001 deaths related to HNSCC^1,2^. The main etiological factors responsible for HNSCC include the consumption of tobacco, betel quid, human papillomavirus (HPV) infection, and immune deficiency^3–8^. The tumors in the oropharyngeal region are primarily associated with HPV infection and show a better survival response to conventional treatment than HPV-negative HNSCC^1,3–9^. Surgical resection, radiotherapy, and chemotherapy constitute the standard HNSCC treatment^1,3–9^. Despite the use of multimodal treatment approaches, HNSCC has a high recurrence rate, and there is an urgent need to identify novel therapeutic targets for its treatment^1,3–9^.

LncRNAs constitute a significant fraction of non-coding RNAs transcribed by the genome and have been recently re-defined as non-coding transcripts exceeding 500 nucleotides^10,11^. They interact with other coding/non-coding RNAs, DNA, and proteins to regulate transcription, RNA and protein stability, translation, post-translational modifications, and epigenetic modifications to regulate cellular functions^10–12^. Many cytoplasmic lncRNAs act as ceRNAs, bind to miRNAs, and promote their target gene expression^10,12,13^. Multiple differentially expressed lncRNAs are associated with different aspects of tumorigenesis such as cell proliferation, migration, invasion, angiogenesis, chemoresistance, and cell death in HNSCC^4,12,14–20^. Recent developments in RNA-based therapeutics have shown immense potential to target deregulated lncRNAs to inhibit cancer progression^21,22^. TGF-β regulates cell proliferation, differentiation, EMT, migration, invasion, chemoresistance, angiogenesis, and apoptosis in many cancers, including HNSCC^4,23–27^. Alterations in TGF-β signaling, such as a decrease in the expression of transforming growth factor-b type II receptor (TGFβRII), SMAD4, and SMAD2, or an increase in the expression of TGF-β1 ligand plays a prominent role in HNSCC pathogenesis^4,25,26,28^.

TGF-β regulates expression of lncRNAs in cancer^27^, and lncRNAs also modulate TGF-β signaling in HNSCC to promote tumorigenesis^4,15–18^. LINC01518 is overexpressed in oesophageal squamous cell carcinoma (ESCC) tissues, and it sponges *miR-1-3p* to promote the PIK3CA/Akt pathway to promote cell proliferation and inhibit apoptosis in ESCC cells^29^. LINC01518 expression is elevated in human glaucoma tissues, and TGF-β induces its expression in human tenon capsule fibroblast (HTF) cells^30^. LINC01518 downregulation significantly suppressed TGF-β1-induced cell proliferation, migration, and autophagy signaling pathway in HTF cells by sponging *miR-216b-5p*^30^. We have recently shown that LINC01518 expression is induced during orthoflavivirus infection, and it interacts with RBM10 and NF-κB to regulate the expression of genes involved in neuroinflammation and endoplasmic stress^31^. However, the function and mechanism of action of LINC01518 in HNSCC were unknown. Here, we show that LINC01518 is overexpressed in high-grade HNSCC tumor samples, and TGF-β induces its expression in HNSCC cells. LINC01518 promotes migration, invasion, and cisplatin resistance in HNSCC by acting as a sponge for *miR-1-3p* and *miR-216b-5p* to promote the expression of their targets Slug and GRP78, respectively. Our results highlight the role of LINC01518 as a potential therapeutic target for HNSCC treatment.

## Materials and methods

### Cell culture and treatments

Human HNSCC cell lines SCC-25 (Oral Squamous cell carcinoma (OSCC) cell line) and FaDu (Hypopharyngeal Cancer cell line) cells were purchased from the American Type Culture Collection (Manassas, VA). SCC-25 cells were cultured in DMEM-F12 (Invitrogen) supplemented with 10% FBS, 2 mM glutamine, and penicillin/streptomycin (Gibco). FaDu cells were cultured in EMEM (Invitrogen) supplemented with 10% FBS, 2 mM glutamine, and penicillin/streptomycin. The cells were maintained at 37°C in a humidified atmosphere containing 5% CO_2_. Serum-starved HNSCC cells were treated with TGF-β1 (PeproTech, #100-21) in a serum-free medium for the dose and duration indicated in the figures and legends. SB505124 (TGFβRI/ALK4/ALK7 inhibitor, Tocris, #3263) was used for pretreatment of HNSCC cells to inhibit the TGF-β signaling wherever indicated^32^.

### Transfection of antisense oligonucleotides (ASOs), miRNA mimics/inhibitors and plasmids

Lipofectamine® RNAiMAX Transfection Reagent (Invitrogen, #13778-075) was used for transfection of ASOs against LINC01518, and miRNA mimic and inhibitor (IN) of *miR-1-3p* and *miR-216b-5p* as per manufacturer’s instructions. ASO/SlugMyc_pcDNA3 and ASO/pcDNA3.1(+)-GRP78/BiP co-transfections were done using lipofectamine 3000 according to the manufacturer’s instructions. The sequences and catalog numbers of ASOs, miRNA mimics and inhibitors used are provided in Supplementary Table I.

### RNA isolation and real-time PCR

RNA isolation and qRT-PCR were performed as described previously^33,34^. All reactions were performed in triplicates and normalized with TBP as an internal control. The 2^−ΔΔCt^ method was used to evaluate the relative gene expression of transcripts. All gene-specific primer sequences are listed in Supplementary Table II.

### Western blot analysis

Protein isolation and western blotting were performed as described previously^33,34^. Briefly, HNSCC cells in culture were washed with PBS and lysed in lysis buffer (1% Triton X, 150 mM NaCl, 10 mM Tris base, 1 mM EDTA, 0.2 mM EGTA, 0.5% IGEPAL, 3 µl/ml protease inhibitor). The BCA method was used to determine the protein concentration, and equal amounts of proteins were separated on SDS-PAGE and transferred to the PVDF membrane. The following primary antibodies were used for western blotting: Slug (1: 2000, CST, #9585), GRP78 (1: 2000, Abcam, #ab21685), Vimentin (1: 2000, CST, #5741), E-Cadherin (1: 2000, CST, #3195), β-actin (1: 100,000, Sigma, #A1978), Secondary antibodies- HRP conjugated anti-rabbit (1: 20,000, Southern Biotech, #4055-05) or anti-mouse IgG (1: 100,000, Invitrogen, #A16072). The protein levels were quantified using ImageJ software (National Institutes of Health, Bethesda, MD).

### Dual-luciferase reporter assay

Reporter assays were performed using the Dual-Luciferase® Reporter Assay System (Promega, #E1910) as described previously^33,34^. To confirm the interaction between LINC01518 and miRNAs, HNSCC cells were co-transfected with the pmirGLO-LINC01518 reporter plasmid and *miR-1-3p*/*miR-216b-5p* mimic or negative control (NC-mimic) using lipofectamine 3000 (Invitrogen, #L3000-015). To confirm the interaction between Slug-3’UTR and *miR-1-3p*, HEK293T cells were co-transfected with pmirGLO-Slug-3’UTR and *miR-1-3p* mimic/NC-mimic. To confirm the interaction between GRP78-3’UTR and *miR-216b-5p*, HEK293T cells were co-transfected with pmirGLO-GRP78-3’UTR and *miR-216b-5p* mimic/NC-mimic. After 36 h of transfection, the cells were lysed and processed for reporter assays.

### Statistical analysis

Results are represented as mean ± SEM unless otherwise stated. We used paired Student’s t-test to compare two experimental groups, and *p* < 0.05 was considered statistically significant. Additional statistical test information is described in the figure legends.

## Results

### LINC01518 expression is up-regulated in HNSCC tissue, and TGF-β induces LINC01518 expression in HNSCC cells

**Fig. 1:**
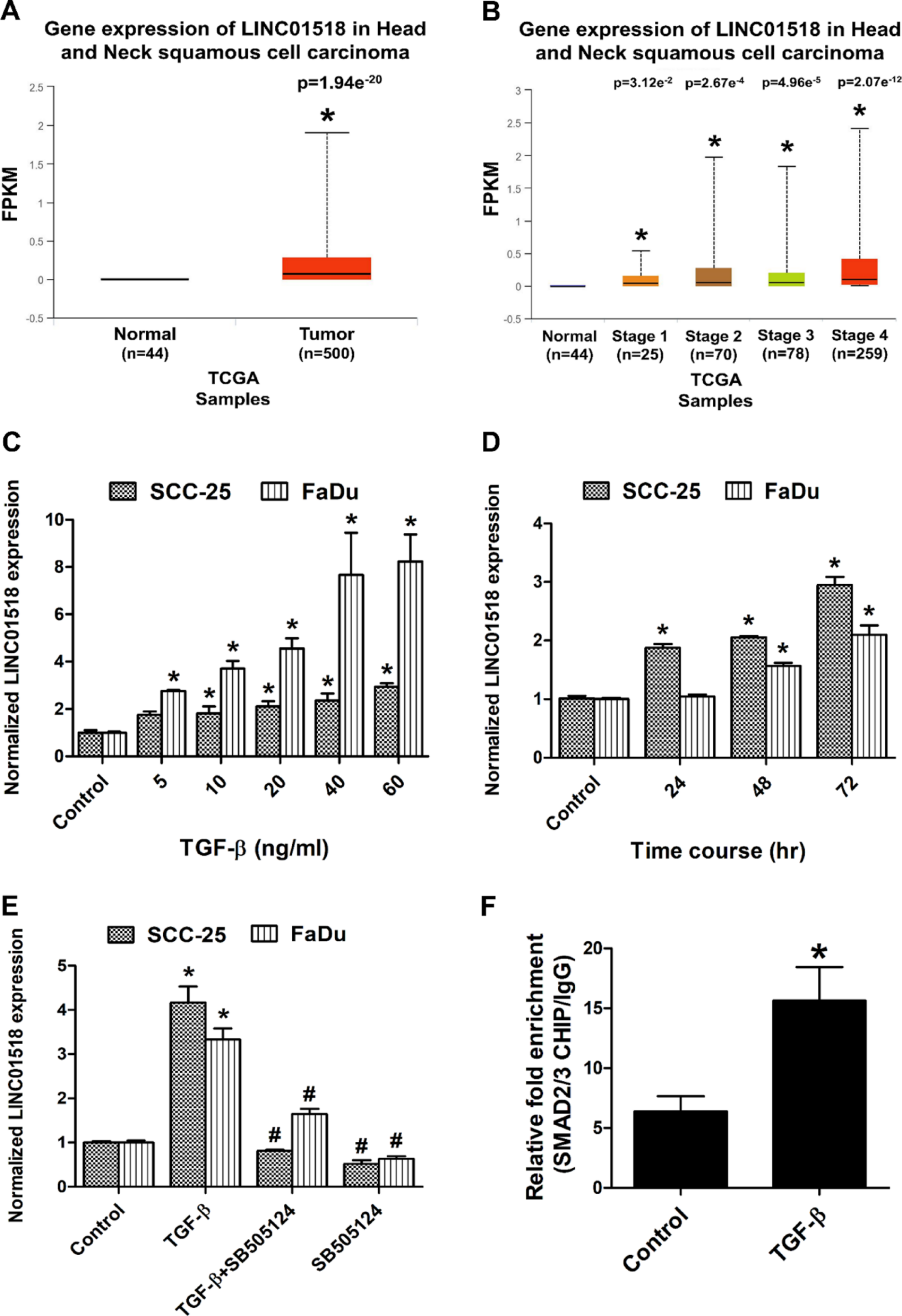
LINC01518 is overexpressed in HNSCC, and TGF-β induces its expression in HNSCC cells. (**A**) LINC01518 expression levels are significantly higher in HNSCC primary tumor samples compared to normal tissues in the TCGA dataset analyzed through UALCAN (*p* value for Normal vs. Tumor is 1.94e^−20^). (**B**) LINC01518 expression levels are elevated in different stages of HNSCC compared to normal tissues in the TCGA dataset analyzed through UALCAN (*p* value for Normal vs. Stage-1 is 3.12e^−2^, Normal vs. Stage-2 is 2.67e^−4^, Normal vs. Stage-3 is 4.96e^−5^, Normal vs. Stage-4 is 2.07e^−12^). (**C**) LINC01518 induction upon TGF-β treatment is dose-dependent. HNSCC cells were treated with the indicated concentration of TGF-β for 48 h, and LINC01518 levels were measured using qRT–PCR. (**D**) LINC01518 is induced upon TGF-β treatment in a time-dependent manner. HNSCC cells were treated with 10 ng/ml of TGF-β for indicated time points, and LINC01518 levels were measured using qRT–PCR. (**E**) TGFβRI inhibitor SB505124 attenuates TGF-β mediated increase in LINC01518 expression. HNSCC cells were pre-treated with 6 µM of SB505124 (TGFβRI/ALK4/ALK7 inhibitor) for 2 h, followed by co-treatment with TGF-β1 (10 ng/ml) for 48 h, and LINC01518 levels were measured using qRT–PCR. (**F**) TGF-β promotes the SMAD2/3 recruitment at the promoter of LINC01518. Relative enrichment of SMAD2/3 at the LINC01518 promoter in control or TGF-β treated SCC-25 cells determined by ChIP-qRT-PCR post 18 h of TGF-β (10 ng/ml) treatment. Enrichment values are relative to Control-IgG. Data information: (For **A**, **B**) *Significant change compared to Normal samples (*p* < 0.05). (For **C**–**F**) Error bars represent the mean ± SEM from three independent experiments. (For **C**–**E**) *Significant change compared to corresponding control (*p* < 0.05). #Significant change compared to TGF-β-treated cells (*p* < 0.05). (For **F**) ChIP purified DNA was analyzed by qRT-PCR, and error bars represent the mean ± SEM from three independent experiments. *Significant change compared to the control (*p* < 0.05). Statistical comparisons were made using the Student’s t-test.

LINC01518 is overexpressed in ESCC and promotes tumorigenesis^29^. However, nothing is known about its role in HNSCC. Hence, we examined the role of LINC01518 in HNSCC pathophysiology by evaluating its expression in HNSCC primary tumor samples from the TCGA dataset using the UALCAN database^35^. LINC01518 expression is significantly higher in HNSCC primary tumor samples as compared to normal tissue (Fig. 1A). Moreover, LINC01518 expression increases with advanced stages of carcinoma in HNSCC (Fig. 1B). Since LINC01518 expression is induced upon TGF-β treatment in HTF cells; hence we asked if TGF-β regulates LINC01518 expression in HNSCC cells^30^. We observed that TGF-β treatment induces LINC01518 expression in a dose-dependent manner in HNSCC cells. LINC01518 expression was increased from ~ 1.8 to ~ 3-fold from 5 to 60 ng/ml of TGF-β in SCC-25 cells, and it increased from ~ 2.7 to ~ 8-fold from 5 to 60 ng/ml of TGF-β in FaDu cells (Fig. 1C). LINC01518 also shows a time-dependent increase in its expression upon TGF-β treatment in HNSCC cells. LINC01518 expression was increased by ~ 2-fold at 24 h and 48 h, followed by ~ 3-fold at 72 h of TGF-β treatment in SCC-25 cells. In FaDu cells, we did not observe a significant change in LINC01518 expression upon TGF-β treatment at 24 h. However, its expression increased from ~ 1.6 fold at 48 h to ~ 2-fold at 72 h of TGF‐β treatment (Fig. 1D).

To confirm the involvement of TGF-β signaling in LINC01518 expression in HNSCC cells, we evaluated LINC01518 expression upon TGF-β treatment in the presence and absence of TGFβRI/ALK4/ALK7 inhibitor SB505124. Inhibition of TGF-β signaling with SB505124 significantly abrogated the TGF-β induced expression of LINC01518 in HNSCC cells (~ 80% and ~ 50% reduction in SCC-25 and FaDu cells, respectively) (Fig. 1E). To confirm the involvement of canonical SMAD2/3 signaling in LINC01518 induction during TGF-β treatment, we performed ChIP-qPCR to determine whether TGF-β promotes the SMAD2/3 binding at the promoter of LINC01518 in SCC-25 cells. ChIP-qPCR revealed increased binding of SMAD2/3 at the promoter of LINC01518 upon TGF-β stimulation as compared to control cells (Fig. 1F). These results suggest that TGF-β promotes LINC01518 expression through the canonical SMAD2/3 signaling in HNSCC.

### Knockdown of LINC01518 reduces cell proliferation, induces apoptosis, and sensitizes HNSCC cells to cisplatin

**Fig. 2:**
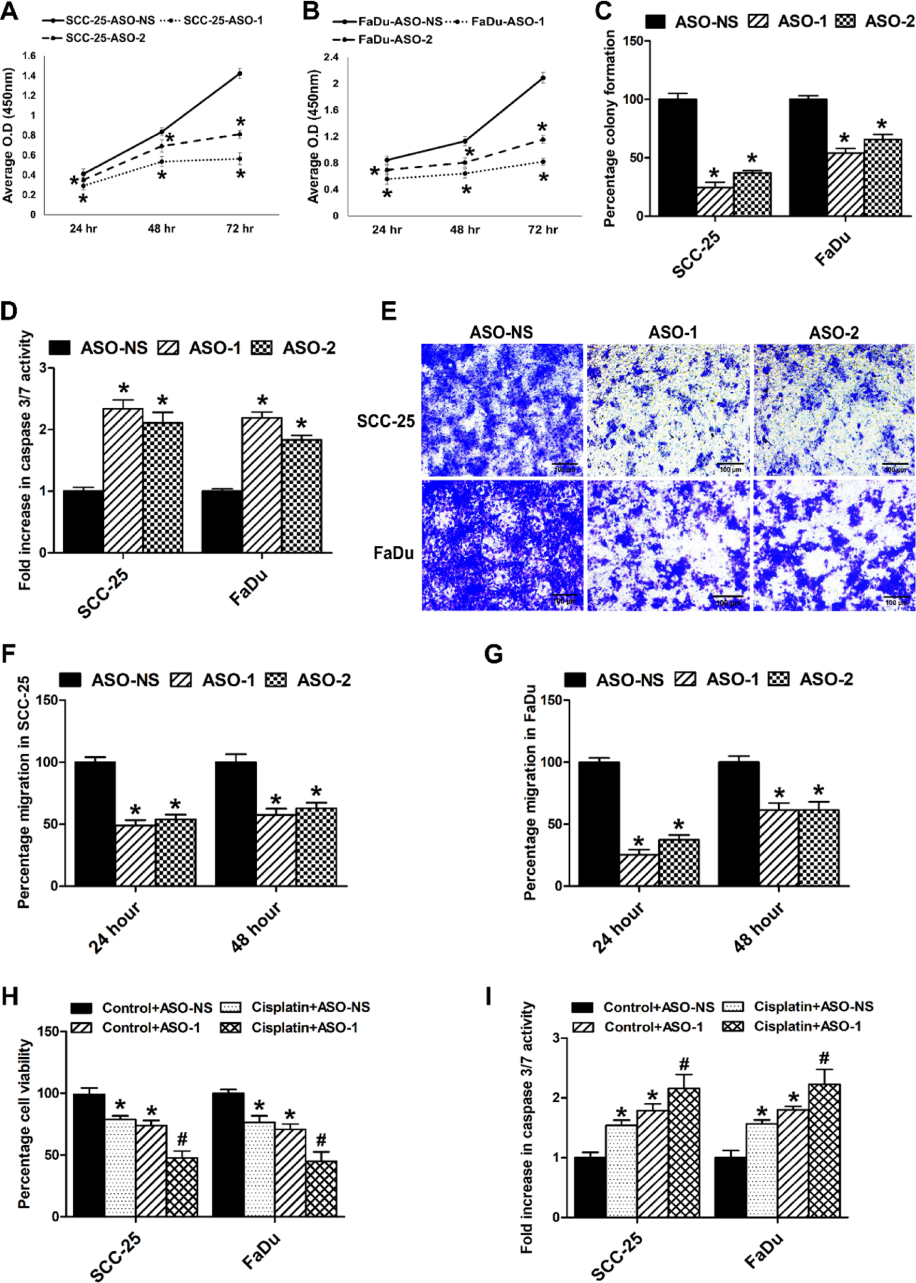
LINC01518 knockdown promotes apoptosis and reduces cell proliferation, migration, and invasion in HNSCC cells. (**A**) SCC-25 cells were transfected with ASO-NS or ASO-1/ASO-2, and cell viability was measured at indicated times using WST-1. (**B**) FaDu cells were transfected with ASO-NS or ASO-1/ASO-2, and cell viability was measured at indicated times using WST-1. (**C**) LINC01518 knockdown reduces the colony formation ability of HNSCC cells. (**D**) LINC01518 knockdown induces apoptosis in HNSCC cells, as indicated by the Caspase 3/7 activity assay. (**E**) Matrigel invasion assay shows that LINC01518 knockdown reduces HNSCC cell invasion. (**F**) Wound healing assay demonstrates reduced SCC-25 cell migration upon LINC01518 knockdown. (**G**) Wound healing assay demonstrates reduced FaDu cell migration upon LINC01518 knockdown. (**H**) LINC01518 knockdown with ASO-1 (25 nM) in combination with cisplatin treatment (10 µM for SCC-25 and 5 µM for FaDu) shows enhanced reduction in HNSCC percentage cell viability and increased sensitivity to cisplatin, as analyzed using WST-1 assay. (**I**) LINC01518 knockdown (25 nM of ASO-1) combined with cisplatin treatment (10 µM for SCC-25 and 5 µM for FaDu) shows enhanced caspase 3/7 activity compared to cisplatin alone. Data information: (For **A-I**) Error bars represent the mean ± SEM from three independent experiments. (For **A**–**G**) *Significant change compared to ASO-NS (*p* < 0.05). (For **H**, **I**) *Significant change compared to Control+ASO-NS treated cells (*p* < 0.05). ^#^Significant change compared to Control+ASO-1 treated cells (*p* < 0.05). Statistical comparisons were made using the Student’s t-test.

To study the physiological function of LINC01518 in HNSCC cells, we established LINC01518 knockdown by using two different ASOs (ASO-1 and ASO-2). LINC01518 knockdown with ASO-1 resulted in a ~ 85% reduction in its expression in both HNSCC cell lines. LINC01518 knockdown with ASO-2 reduced its expression by ~ 65% in SCC-25 cells and ~ 80% in FaDu cells (Fig. S1A). LINC01518 knockdown reduced cell proliferation in SCC-25 cells by ~ 25% at 24 h and 48 h time points and ~ 52% at 72 h (Fig. 2A). LINC01518 depletion in FaDu cells reduced cell proliferation by ~ 26% at 24 h, ~ 35% at 48 h, and ~ 53% at 72 h (Fig. 2B). LINC01518 knockdown also results in a significant decrease in colony formation of SCC-25 cells by ~ 74% with ASO-1 and ~ 63% with ASO-2 compared to cells transfected with ASO-NS. We also observed a ~ 45% and ~ 34% decrease in colony formation of FaDu cells upon LINC01518 depletion with ASO-1 and ASO-2, respectively (Fig. 2C, S1B & S1C). LINC01518 depletion also results in a ~ 2-fold increase in caspase 3/7 activity in HNSCC cells (Fig. 2D). In addition, LINC01518 knockdown results in a ~ 60% and ~ 50% reduction in the cell invasion of SCC-25 and FaDu cells, respectively (Fig. 2E & S1D). Wound healing assay in SCC-25 cells after LINC01518 knockdown show ~ 50% and ~ 40% reduction in cell migration after 24 h and 48 h time points, respectively (Fig. 2F & S1E). In FaDu cells, LINC01518 knockdown resulted in ~ 69% and ~ 38% decrease in cell migration after 24 h and 48 h time points, respectively (Fig. 2G & S1F). Cisplatin is the primary choice of drug used to treat HNSCC^6,19,36^. Hence, we evaluated the effect of LINC01518 knockdown on cisplatin sensitivity in HNSCC cells. Cisplatin treatment with LINC01518 knockdown using ASO-1 (25 nM) resulted in a significantly reduced cell proliferation in HNSCC cells compared to cells treated with cisplatin and ASO-NS (Fig. 2H). In addition, cisplatin treatment with LINC01518 depletion resulted in a ~ 1.4-fold increase in caspase 3/7 activity in HNSCC cells compared to cells treated with cisplatin and ASO-NS (Fig. 2I). Collectively, these results indicate that LINC01518 acts as an oncogene to promote proliferation, migration, invasion, and cisplatin resistance in HNSCC cells.

### LINC01518 regulates the expression of a subset of TGF-β target genes

**Fig. 3:**
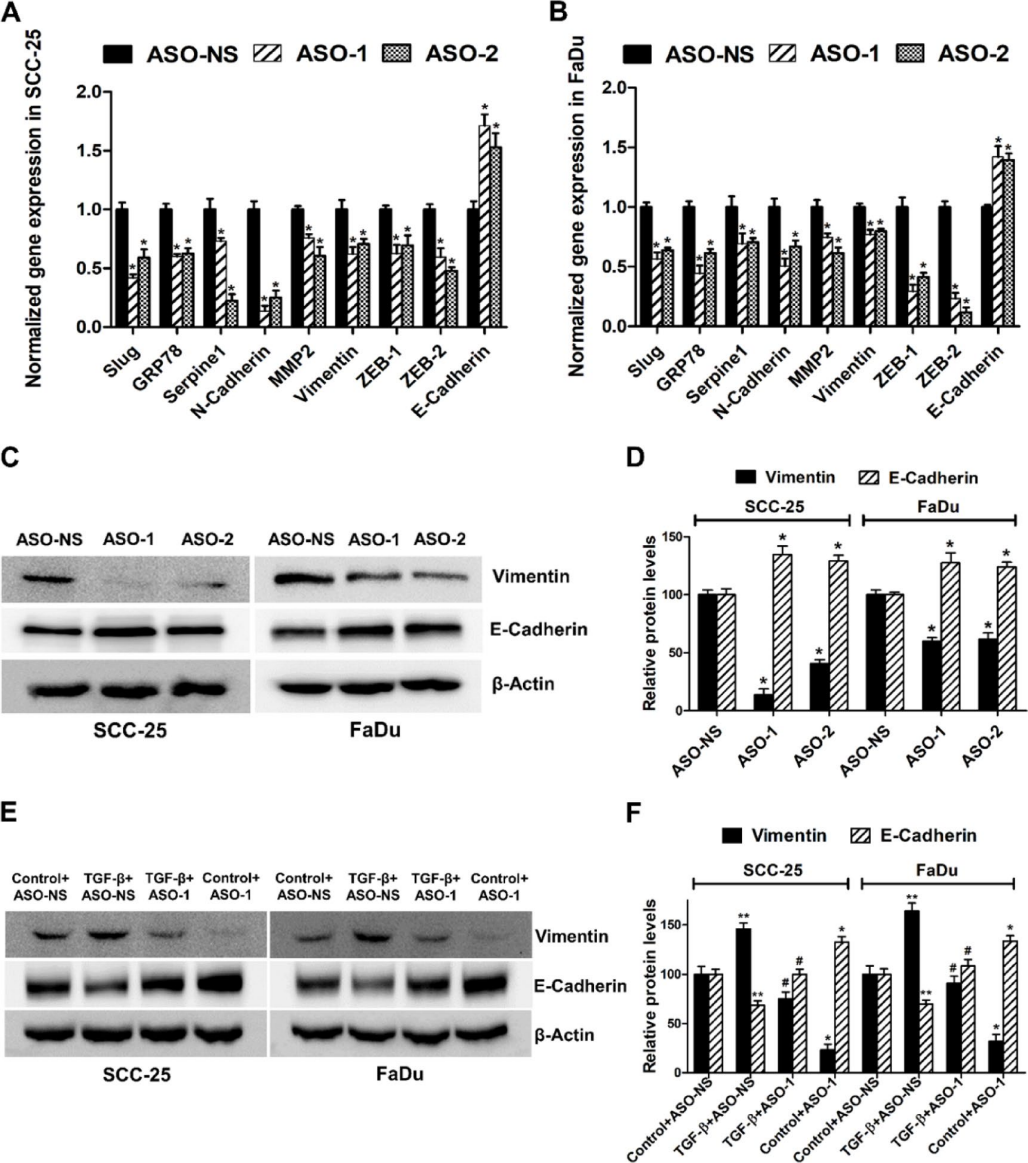
LINC01518 regulates the expression of a subset of TGF-β target genes in HNSCC cells. (**A**) SCC-25 cells transfected with ASO-NS or ASO-1/ASO-2, and transcript levels of indicated genes were measured 48 h post-transfection using qRT–PCR. (**B**) FaDu cells transfected with ASO-NS or ASO-1/ASO-2, and transcript levels of indicated genes were measured 48 h post-transfection using qRT–PCR. (**C**) Western blot analysis of Vimentin and E-Cadherin upon LINC01518 knockdown in HNSCC cells. SCC-25 and FaDu cells were transfected with ASO-NS or ASO-1/ASO-2, and protein levels of indicated genes were measured 48 h post-transfection. A representative blot from three independent experiments with similar results is shown. Blots were re-probed for β-actin to establish equivalent loading. (**D**) Quantification of western blots of indicated proteins shown in (**C**). (**E**) Analysis of Vimentin and E-Cadherin expression with or without LINC01518 knockdown during TGF-β treatment of HNSCC cells. Cells were transfected with ASO-NS or ASO-1, and twenty-four hours after transfection, treated with or without TGF-β (10 ng/ml for 48 h), and protein levels of indicated genes were measured. A representative blot is shown from three independent experiments with similar results. Blots were re-probed for β-actin to establish equivalent loading. (**F**) Quantification of western blots of indicated proteins shown in (**E**). Data information: (For **A**–**F**) Error bars represent the mean ± SEM from three independent experiments. (For **A**–**D**) *Significant change compared to ASO-NS (*p* < 0.05). (For **F**) **Significant change compared to Control+ASO-NS cells (*p* < 0.05). ^#^Significant change compared to TGF-β+ASO-NS cells (*p* < 0.05). *Significant change compared to Control+ASO-NS cells (*p* < 0.05). Statistical comparisons were made using the Student’s t-test.

Since TGF-β promotes LINC01518 expression in HNSCC cells, we evaluated the expression of the TGF-β gene ontology group upon LINC01518 depletion. Knockdown of LINC01518 results in a significant downregulation of Slug, GRP78, Serpine1, N-Cadherin, MMP2, Vimentin, ZEB-1, and ZEB-2 expression in HNSCC cells (Fig. 3A,B). In addition, LINC01518 depletion also results in a significant upregulation of the epithelial marker E-Cadherin (Fig. 3A,B). Several other TGF-β-regulated genes, such as TIMP2, CTGF, Twist, TGFB1, TGFβR1, and Snail, showed no significant change in expression upon LINC01518 knockdown in FaDu cells (Fig. S2). Since TGF-β induces EMT, and LINC01518 regulates the expression of TGF-β target genes, we checked the impact of LINC01518 depletion on the TGF-β induced EMT by evaluating the expression of Vimentin (mesenchymal marker) and E-Cadherin (epithelial marker). In agreement with the transcript data, LINC01518 silencing results in a ~ 56% decrease in Vimentin and ~ 29% increase in E-Cadherin protein levels in HNSCC cells (Fig. 3C,D). Moreover, LINC01518 depletion significantly attenuates TGF-β mediated increase in the Vimentin expression and decrease in the E-Cadherin expression (Fig. 3E,F). These results suggest that LINC01518 regulates the expression of Slug, GRP78, Serpine1, N-Cadherin, MMP2, Vimentin, ZEB-1, ZEB-2, and E-Cadherin genes to promote EMT in SCC-25 and FaDu cells.

### LINC01518 acts as a ceRNA for *miR-1-3p* and *miR-216b-5p*, and their expression is downregulated in HNSCC

**Fig. 4:**
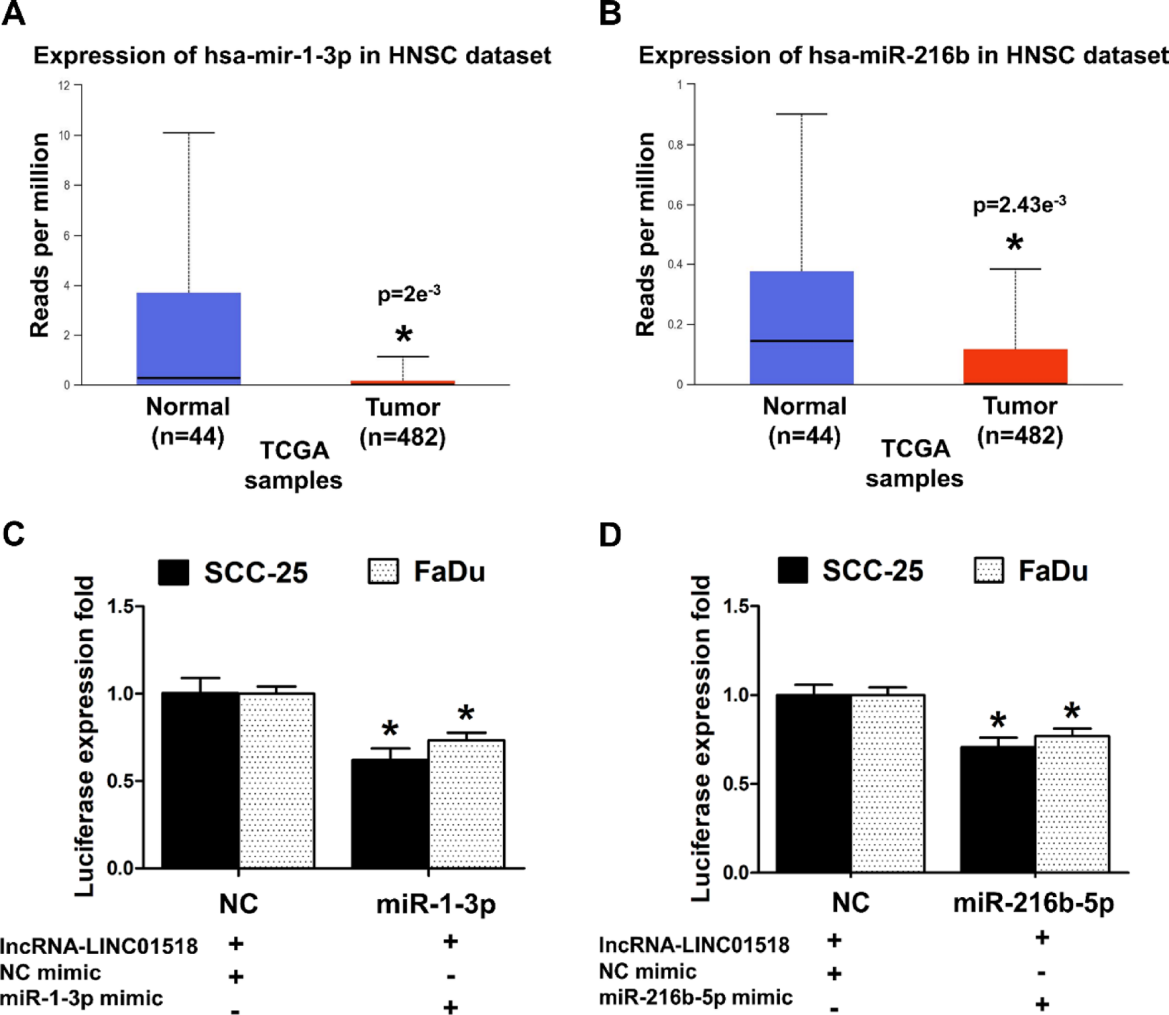
*LINC01518* acts as a ceRNA to sponge *miR-1-3p* and *miR-216b-5p,* and their levels are reduced in HNSCC. (**A**) *MiR-1-3p* expression levels are significantly lower in HNSCC primary tumor samples compared to normal tissues in the TCGA dataset analyzed through UALCAN. (*p* value for Normal vs. Tumor is 2e^−3^). (**B**) *MiR-216b-5p* expression levels are significantly lower in HNSCC primary tumor samples compared to normal tissues in the TCGA dataset analyzed through UALCAN. (*p* value for Normal vs. Tumor is 2.43e^−3^). (**C**) Luciferase activity assay suggests that LINC01518 interacts with *miR-1-3p*; relative luciferase activity was measured in HNSCC cells co-transfected with *miR-1-3p* mimic/NC-mimic and pmiRGLO-LINC01518 construct. Luminescence signals were measured 36 h post-transfection using dual luciferase assay. (**D**) Luciferase activity assay suggests that LINC01518 interacts with *miR-216b-5p*; relative luciferase activity was measured in HNSCC cells co-transfected with *miR-216b-5p* mimic/NC-mimic and pmiRGLO-LINC01518 construct. Luminescence signals were measured 36 h post-transfection using dual luciferase assay. Data information: (For **A**, **B**) *Significant change compared to Normal samples (*p* < 0.05). (For **C**, **D**) Error bars represent the mean ± SEM from three independent experiments. *Significant change compared to NC-mimic (*p* < 0.05). Statistical comparisons were made using the Student’s t-test.

Several lncRNAs function as endogenous miRNA sponges to promote miRNA target gene expression^10,12,13^. LINC01518 is known to bind to *miR-1-3p* and *miR-216b-5p*^29,30^. Hence, we decided to explore the role of *miR-1-3p* and *miR-216b-5p* in the context of LINC01518 in HNSCC pathogenesis. Firstly, we evaluated the expression of these miRNAs in HNSCC patient samples using the UALCAN database. We observed that the expression of *miR-1-3p* and *miR-216b-5p* is significantly lower in HNSCC primary tumor samples compared to normal tissue (Fig. 4A,B). Next, we decided to confirm the interaction of LINC01518 with *miR-1-3p* and *miR-216b-5p* using dual luciferase assay in HNSCC cells. For this, we cloned the full-length LINC01518 sequence in the pmirGLO vector downstream of the firefly luciferase gene. Cells co-transfected with pmirGLO-LINC01518 reporter and *miR-1-3p* mimic had ~ 33% less reporter activity compared to the control cells co-transfected with pmirGLO-LINC01518 and NC-mimic (Fig. 4C). Likewise, cells co-transfected with pmirGLO-LINC01518 reporter and *miR-216b-5p* mimic had ~ 27% less reporter activity compared to the control cells co-transfected with pmirGLO-LINC01518 and NC-mimic (Fig. 4D). These results confirm that LINC01518 binds to *miR-1-3p* and *miR-216b-5p* in HNSCC cells.

### LINC01518 functions as a miRNA sponge for *miR-1-3p* to regulate the expression of Slug in HNSCC

**Fig. 5:**
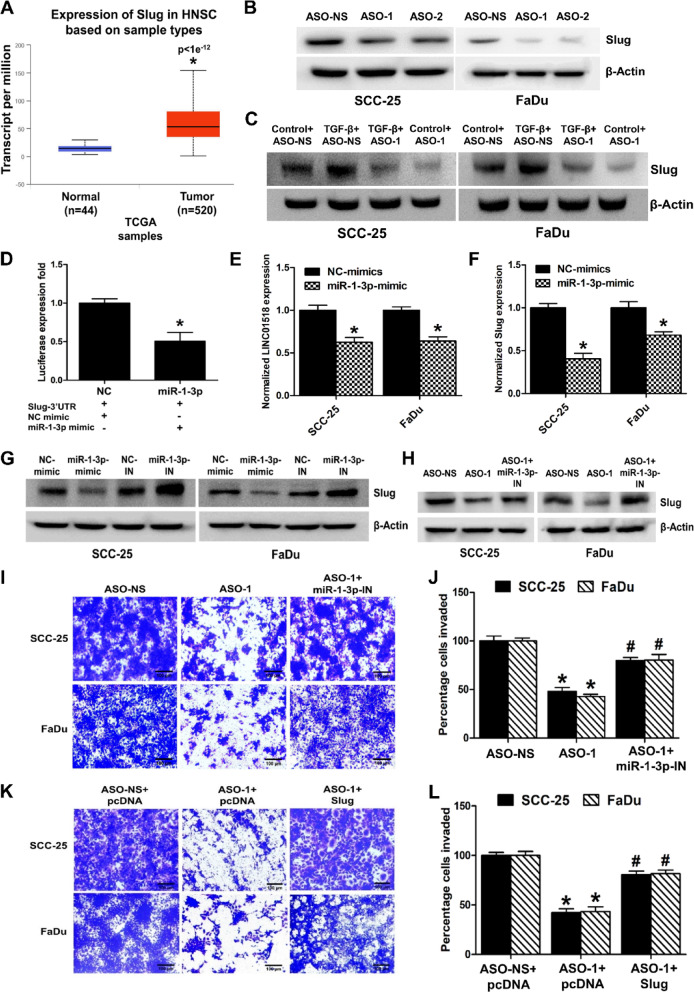
LINC01518 sponges *miR-1-3p* to regulate Slug expression in HNSCC. (**A**) Slug expression is significantly higher in HNSCC primary tumor samples compared to normal tissues in the TCGA dataset analyzed through UALCAN (*p* value for Normal vs. Tumor is < 1e^−12^). (**B**) Western blot analysis of Slug post-LINC01518 knockdown in HNSCC cells. Cells were transfected with ASO-NS or ASO-1/ASO-2, and Slug protein levels were measured 48 h post-transfection. A representative blot is shown from three independent experiments with similar results. Blots were re-probed for β-actin to establish equivalent loading. (**C**) Western blot analysis of Slug upon LINC01518 knockdown during TGF-β treatment of HNSCC cells. Cells were transfected with ASO-NS or ASO-1 and twenty-four hours after transfection, treated with or without TGF-β (10 ng/ml for 48 h), and Slug protein levels were measured. A representative blot is shown from three independent experiments with similar results. Blots were re-probed for β-actin to establish equivalent loading. (**D**) Luciferase activity assay demonstrates that *miR-1-3p* interacts with Slug-3’UTR; relative luciferase activity was measured in HEK293T cells co-transfected with *miR-1-3p* mimic/NC-mimic and pmiRGLO-Slug-3’UTR construct. Luminescence signals were measured 36 h post-transfection using dual luciferase assay. (**E**) Downregulation of LINC01518 transcript levels upon *miR-1-3p* overexpression measured by qRT-PCR in HNSCC cells. (**F**) Downregulation of Slug transcript levels upon *miR-1-3p* overexpression measured by qRT-PCR in HNSCC cells. (**G**) Slug protein levels upon transfection with *miR-1-3p* mimic and inhibitor. HNSCC cells transfected with 80 nM of NC-mimic/NC-inhibitor/*miR-1-3p* mimic/*miR-1-3p* inhibitor and Slug protein levels were analyzed 48 h post-transfection using western blotting. A representative blot is shown from three independent experiments with similar results. Blots were re-probed for β-actin to establish equivalent loading. (**H**) Rescue of loss of Slug expression due to LINC01518 depletion upon *miR-1-3p* inhibition in HNSCC cells. A representative blot is shown from three independent experiments with similar results. Blots were re-probed for β-actin to establish equivalent loading. (**I**) Representative image of the rescue of invasion due to LINC01518 depletion upon *miR-1-3p* inhibition in HNSCC cells. (**J**) Quantification of invasion upon LINC01518 knockdown and *miR-1-3p* inhibition shown in (**I**). (**K**) Representative image of the rescue of invasion due to LINC01518 depletion upon Slug overexpression in HNSCC cells. (**L**) Quantification of invasion upon LINC01518 knockdown and Slug overexpression shown in (**K**). Data information: (For **A**) *Significant change compared to Normal samples (*p* < 0.05). (For **D**–**L**) Error bars represent the mean ± SEM from three independent experiments. (For **D**–**F**) *Significant change compared to NC-mimic (*p* < 0.05). (For **J**) *Significant change compared to ASO-NS (*p* < 0.05). #Significant change compared to ASO-1 (*p* < 0.05). (For **L**) *Significant change compared to ASO-NS+pcDNA (*p* < 0.05). #Significant change compared to ASO-1+pcDNA (*p* < 0.05). Statistical comparisons were made using the Student’s t-test.

Since LINC01518 interacts with *miR-1-3p* (Fig. 4C), we searched which of the LINC01518 target genes we identified (Fig. 3A,B) have *miR-1-3p* binding sites in their 3’UTR*.* Targetscan analysis revealed Slug as a target gene of *miR-1-3p;* interestingly, other than Slug, no other LINC01518 target genes shown in Fig. 3A &B were found to interact with *miR-1-3p* using Targetscan^37^. Furthermore, Peng et al. have also demonstrated that *miR-1-3p* targets Slug in oral cancer^38^. Hence, we decided to evaluate the role of LINC01518 and *miR-1-3p*/Slug axis in HNSCC pathogenesis. First, we assessed the expression of Slug in HNSCC primary tumor samples from the TCGA dataset. The expression of Slug transcript is significantly increased in HNSCC primary tumor samples as compared to normal tissue (Fig. 5A), which is consistent with LINC01518 overexpression and *miR-1-3p* downregulation in HNSCC samples (Fig. 1A  & 4A). In addition, the Kaplan–Meier survival analysis demonstrates a poor overall survival of HNSCC patients with high Slug expression (Fig. S3A). Since LINC01518 knockdown results in the downregulation of Slug transcript (Fig. 3A,B), we evaluated the impact of LINC01518 knockdown on Slug protein levels. In agreement with the transcript data, LINC01518 silencing results in a ~ 44% decrease in Slug protein levels in HNSCC cells (Fig. 5B & S3B). Moreover, LINC01518 depletion attenuates the TGF-β mediated increase in Slug expression in SCC-25 and FaDu cells (Fig. 5C & S3C). To validate the interaction of *miR-1-3p* and Slug, we cloned the 3’UTR region of Slug in the pmirGLO vector downstream of the firefly luciferase gene. HEK293T cells co-transfected with pmirGLO-Slug-3’UTR reporter and *miR-1-3p* mimic had ~ 50% less reporter activity compared to control cells co-transfected with pmirGLO-Slug-3’UTR reporter and NC-mimic (Fig. 5D).

To evaluate the effect of *miR-1-3p* on LINC01518 and Slug expression, we determined their transcript levels upon *miR-1-3p* overexpression. We observed ~ 36% reduction in LINC01518 expression upon treatment with *miR-1-3p* mimic in HNSCC cells (Fig. 5E). We also observed ~ 60% and ~ 32% reduction in Slug transcript levels after *miR-1-3p* overexpression in SCC-25 and FaDu cells, respectively (Fig. 5F). Consistent with this, transfection of *miR-1-3p* mimic significantly reduced Slug protein levels by ~ 45% and knockdown of *miR-1-3p* using miRNA inhibitor increased Slug protein levels by ~ 43% in HNSCC cells (Fig. 5G & S3D). Given that *miR-1-3p* targets LINC01518 and Slug, and because we observed downregulation of Slug upon LINC01518 depletion, we asked whether LINC01518 could sponge *miR-1-3p* to stabilize Slug expression. We observed that the decrease in Slug protein levels due to LINC01518 depletion was partially rescued upon combined depletion of LINC01518 and *miR-1-3p* (Fig. 5H & S3E). Invasion is one of the primary hallmarks of HNSCC^7^. Since LINC01518 acts as a ceRNA for *miR-1-3p* to promote Slug expression, we asked whether *miR-1-3p* inhibition or Slug overexpression can rescue the LINC01518 inhibition-mediated decrease in HNSCC cell invasion. Both *miR-1-3p* inhibition and Slug overexpression in HNSCC cells partially rescue the reduction in invasion due to LINC01518 knockdown (Fig. 5I–L). These results indicate that LINC01518 sponges *miR-1-3p* to promote Slug expression and invasion in HNSCC cells.

### LINC01518 functions as a miRNA sponge for *miR-216b-5p* to regulate the expression of GRP78 in HNSCC

**Fig. 6:**
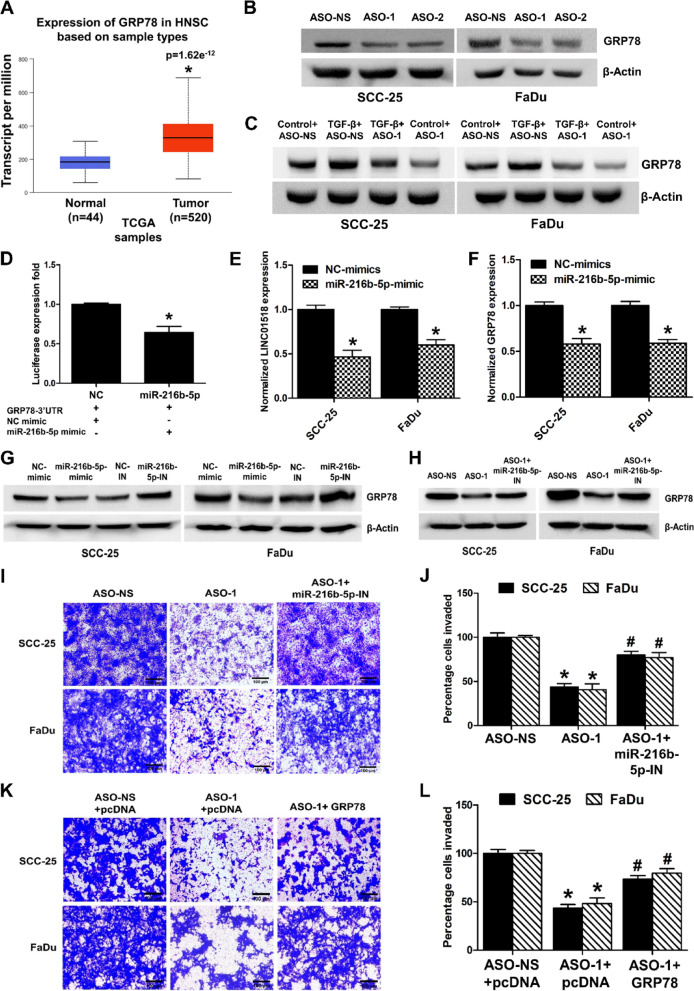
LINC01518 sponges *miR-216b-5p* to regulate GRP78 expression in HNSCC. (**A**) GRP78 expression is significantly higher in HNSCC primary tumor samples than normal tissues in the TCGA dataset analyzed through UALCAN (*p* value for Normal vs. Tumor is 1.62e^−12^). (**B**) Western blot analysis of GRP78 upon LINC01518 knockdown in HNSCC cells. Cells were transfected with ASO-NS or ASO-1/ASO-2, and GRP78 protein levels were measured 48 h post-transfection. A representative blot is shown from three independent experiments with similar results. Blots were re-probed for β-actin to establish equivalent loading. (**C**) Western blot analysis of GRP78 upon LINC01518 knockdown during TGF-β treatment of HNSCC cells. Cells were transfected with ASO-NS or ASO-1 and twenty-four hours after transfection, treated with or without TGF-β (10 ng/ml for 48 h), and GRP78 protein levels were measured. A representative blot is shown from three independent experiments with similar results. Blots were re-probed for β-actin to establish equivalent loading. (**D**) Luciferase activity assay demonstrates that *miR-216b-5p* interacts with GRP78-3’UTR; relative luciferase activity was measured in HEK293T cells co-transfected with *miR-216b-5p* mimic/NC-mimic and pmiRGLO-GRP78-3’UTR construct. Luminescence signals were measured 36 h post-transfection using dual luciferase assay. (**E**) Downregulation of LINC01518 transcript levels upon *miR-216b-5p* overexpression measured by qRT-PCR in HNSCC cells. (**F**) Downregulation of GRP78 transcript levels upon *miR-216b-5p* overexpression measured by qRT-PCR in HNSCC cells. (**G**) GRP78 protein levels upon transfection with *miR-216b-5p* mimic and inhibitor. HNSCC cells transfected with 80 nM of NC-mimic/NC-inhibitor/*miR-216b-5p* mimic/*miR-216b-5p* inhibitor and GRP78 protein levels were analyzed 48 h post-transfection using western blotting. A representative blot is shown from three independent experiments with similar results. Blots were re-probed for β-actin to establish equivalent loading. (**H**) Rescue of loss of GRP78 expression due to LINC01518 depletion upon *miR-216b-5p* inhibition in HNSCC cells. A representative blot is shown from three independent experiments with similar results. Blots were re-probed for β-actin to establish equivalent loading. (**I**) Representative image of the rescue of invasion due to LINC01518 depletion upon *miR-216b-5p* inhibition in HNSCC cells. (**J**) Quantification of invasion upon LINC01518 knockdown and *miR-216b-5p* inhibition shown in (**I**). (**K**) Representative image of the rescue of invasion due to LINC01518 depletion upon GRP78 overexpression in HNSCC cells. (**L**) Quantification of invasion upon LINC01518 knockdown and GRP78 overexpression shown in (**K**). Data information: (For **A**) *Significant change compared to Normal samples (*p* < 0.05). (For **D**–**L**) Error bars represent the mean ± SEM from three independent experiments. (For **D**–**F**) *Significant change compared to NC-mimic (*p* < 0.05). (For **J**) *Significant change compared to ASO-NS (*p* < 0.05). #Significant change compared to ASO-1 (*p* < 0.05). (For **L**) *Significant change compared to ASO-NS+pcDNA (*p* < 0.05). #Significant change compared to ASO-1+pcDNA (*p* < 0.05). Statistical comparisons were made using the Student’s t-test.

Since LINC01518 also interacts with *miR-216b-5p* (Fig. 4D), we searched which of the LINC01518 target genes identified by us (Fig. 3A,B) have *miR-216b-5p* binding sites in their 3’UTR. To our surprise, Targetscan analysis did not identify any of the genes regulated by LINC01518 (Fig. 3A,B) as *miR-216-5p* targets. However, miRWalk analysis revealed an interaction between *miR-216b-5p* and GRP78^39^. Moreover, our group has recently shown that *miR-216b-5p* targets GRP78 to promote JEV and WNV replication and associated cell death^40^. Therefore, we evaluated the role of LINC01518 and *miR-216b-5p*/GRP78 axis in HNSCC pathogenesis. First, we assessed the expression of GRP78 in HNSCC primary tumor samples from the TCGA dataset. Like Slug, the expression of GRP78 is significantly increased in HNSCC primary tumor samples as compared to normal tissue (Fig. 6A), which is consistent with LINC01518 overexpression and *miR-216b-5p* downregulation in HNSCC samples (Fig. 1A and 4B). Moreover, HNSCC patients with high GRP78 expression show poor overall survival (Fig. S4A). First, we evaluated the impact of LINC01518 depletion on GRP78 protein levels. In agreement with the transcript data, LINC01518 silencing results in a ~ 40% decrease in GRP78 protein levels in HNSCC cells (Fig. 6B & S4B). Moreover, LINC01518 depletion attenuates the TGF-β mediated increase in GRP78 protein expression in SCC-25 and FaDu cells (Fig. 6C & S4C). Next, to validate the interaction of *miR-216b-5p* and GRP78, we cloned the 3’UTR region of GRP78 in the pmirGLO vector downstream of the firefly luciferase gene. HEK293T cells co-transfected with pmirGLO-GRP78-3’UTR reporter and *miR-216b-5p* mimic had ~ 36% less reporter activity compared to cells co-transfected with pmirGLO-GRP78-3’UTR reporter and NC-mimic (Fig. 6D).

Next, we determined the effect of *miR-216b-5p* overexpression on LINC01518 and GRP78 expression in HNSCC cells. We observed ~ 54% and ~ 40% reduction in LINC01518 expression upon *miR-216b-5p* overexpression in SCC-25 and FaDu cells, respectively (Fig. 6E). *MiR-216b-5p* overexpression also resulted in ~ 42% reduction in GRP78 transcript levels in HNSCC cells (Fig. 6F). Likewise, the transfection of *miR-216b-5p* mimic resulted in ~ 32% reduction and *miR-216b-5p* inhibitor resulted in ~ 44% increase in GRP78 protein levels in HNSCC cells (Fig. 6G & S4D). Moreover, the downregulation in GRP78 protein levels due to LINC01518 knockdown was partially rescued upon combined depletion of LINC01518 and *miR-216b-5p* in HNSCC cells (Fig. 6H & S4E). Next, we checked whether *miR-216b-5p* inhibition or GRP78 overexpression can rescue the LINC01518 knockdown mediated reduction in HNSCC cell invasion. We show that *miR-216b-5p* inhibition or GRP78 overexpression partially rescues the decrease of invasion due to LINC01518 knockdown in HNSCC cells (Fig. 6I–L). These results indicate that LINC01518 sponges *miR-216b-5p* to promote GRP78 expression and invasion in HNSCC.

## Discussion

HNSCC is emerging as one of the most common cancer types worldwide, and its incidence rate will increase by 30% in 2030^3,41,42^. The prognosis of HNSCC is poor due to its resistance to chemo, radio, immuno-, and targeted therapy^3,7,8,43,44^. Therefore, there is an urgent need to discover novel pathways for therapeutic targeting in HNSCC. Altered TGF-β signaling is associated with increased EMT, tumor growth, and drug resistance in HNSCC^4,28^. Fresolimumab, which targets all human isoforms of TGF-β, has shown significant results in treating multiple cancers^45,46^. Small-molecule inhibitors targeting TGFβRI, such as LY3200882 and PF-06952229, have been evaluated in phase I clinical trials that included patients with HNSCC^47,48^. LncRNAs have emerged as pervasive regulators of multiple cancer hallmarks such as proliferation, apoptosis, invasion, and metastasis in HNSCC^14,22^. TGF-β regulates the expression of lncRNAs in HNSCC to promote tumorigenesis, and changes in lncRNAs also modulate the TGF-β pathway to enhance cancer development^4^. Recent advances in genome editing, oligonucleotide chemistry, and RNA engineering are leading the way for efficient and cost-effective lncRNA-focused drug discovery pipelines^21^. Therefore, exploring the role of TGF-β regulated lncRNAs as potential therapeutic targets for HNSCC is pertinent.

LINC01518 expression is up-regulated in ESCC tissues, and it promotes the PIK3CA/Akt pathway by sponging *miR-1-3p* to increase proliferation and inhibit apoptosis^29^. However, the role of LINC01518 in HNSCC pathogenesis was unknown. We show that LINC01518 expression is elevated in high-grade HNSCC tumor samples (Fig. 1A,B). TGF-β induces the expression of LINC01518 in HTF cells^30^. In line with this, we show that TGF-β induces the expression of LINC01518 in HNSCC cells using the canonical SMAD2/3 signaling (Fig. 1C–F).

LncRNAs contribute to TGF-β-mediated invasion, metastasis, and EMT in multiple cancers, including HNSCC. TGF-β induces the expression of lncRNA MALAT-1 in HNSCC cells, and it promotes invasion, migration, and tumor growth by sponging *miR-30a*^15^. TGF-β promotes the expression of lncRNA MIR155HG and *miR-155-5p* in laryngeal squamous cell carcinoma (LSCC), which synergistically promotes proliferation, migration, and invasion by targeting SOX-10^16^. LncRNA UCA-1 expression is induced upon TGF-β treatment in tongue cancer cells, and it promotes EMT and invasion by sponging *miR*-*124* to promote JAG1/Notch1 signaling^17^. Similar to these TGF-β regulated lncRNAs, LINC01518 knockdown in HNSCC cells promotes apoptosis and reduces proliferation, colony formation, migration, invasion, and TGF-β induced EMT. Furthermore, LINC01518 depletion sensitizes HNSCC cells to cisplatin-mediated cell death (Fig. 2). LINC01518 knockdown in HNSCC cells also results in downregulation of subset of TGF-β target genes such as Slug, GRP78, Serpine1, N-Cadherin, MMP2, Vimentin, ZEB-1, and ZEB-2 and promotes the expression of epithelial marker, E-Cadherin in HNSCC (Fig. 3).

LINC01518 sequesters *miR-1-3p* to promote PIK3CA/Akt signaling^29^. LINC01518 also sponges *miR-216b-5p* in TGF-β1 treated HTF cells to promote autophagy, but the genes targeted by *miR-216b-5p* in these cells are unclear^30^. Interestingly, the expression of both *miR-1-3p* and *miR-216b-5p* is downregulated in HNSCC tissues^49,50^. We show that LINC01518 acts as a ceRNA for *miR-1-3p* and *miR-216b-5p* in HNSCC cells to promote the expression of Slug and GRP78, respectively (Figs. 4, 5 and 6).

Slug is a transcription factor of the Snail family, which acts as an effector of the TGF-β induced EMT, invasion, metastasis, and disease recurrence in HNSCC^51–57^. Slug levels are up-regulated in HNSCC, and its high expression correlates with poor overall survival in HNSCC patients^51,57,58^. Peng et al. have demonstrated that *miR-1-3p* targets Slug to suppress oral cancer^38^. Apart from Slug, *miR-1-3p* is also known to target ITGB4, DKK1, EGFR, c-MET, TAGLN2, Fibronectin-1, PNP, and PTMA genes to inhibit proliferation, migration, and invasion in HNSCC^38,49,59–61^. We show that LINC01518 knockdown reduces TGF-β mediated increase in EMT and Slug expression (Figs. 3E,F, 5C, S3C).

**Fig. 7:**
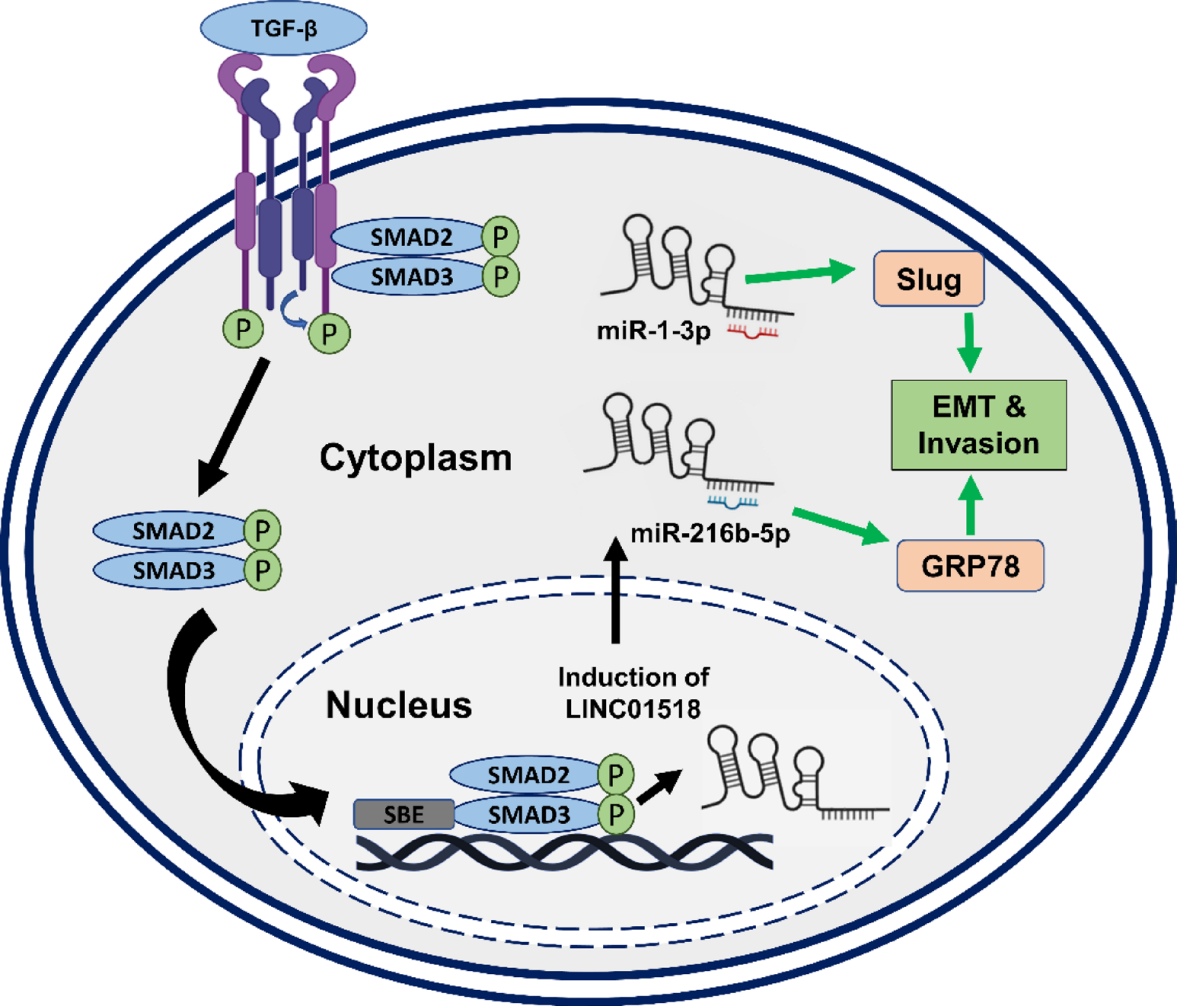
A working model of LINC01518 function in HNSCC. LINC01518 expression is induced by the TGF-β pathway. It sequesters *miR-1-3p* and *miR-216b-5p* from binding their targets Slug and GRP78, respectively. This results in enhanced Slug and GRP78 expression, which promotes invasion and EMT in HNSCC.

TGF-β induces GRP78 expression, and it promotes the TGF-β1 secretion by activating the TGF-β/SMAD2/3 signaling to promote EMT and migration^62–64^. GRP78 plays crucial roles during cancer development by increasing cell survival, stemness, EMT, migration, invasion, and therapy resistance in HNSCC, and its high expression correlates with poor overall survival in HNSCC patients^65,66^. We show that GRP78 is a target gene of *miR-216b-5p,* which binds to LINC01518. Interestingly, *miR-216b* inhibits the proliferation, invasion, and tumor growth in nasopharyngeal carcinoma by targeting KRAS and PKCα^67,68^. We show that LINC01518 knockdown reduces TGF-β mediated increase in GRP78 expression. LINC01518 acts as ceRNA for *miR-216b-5p* to promote GRP78 expression (Fig. 6). Overall, our findings suggest that TGF-β fosters the transcription of LINC01518 in HNSCC cells and it regulates *miR-1-3p*/Slug and *miR-216b-5p*/GRP78 axis to promote EMT in HNSCC (Fig. 7). However, the mechanism of LINC01518 mediated regulation of Serpine1, N-Cadherin, MMP2, Vimentin, ZEB-1, ZEB-2, and E-Cadherin needs further investigation.

HNSCC includes multiple subtypes, such as OSCC, Hypopharyngeal cancer, Oropharyngeal cancer, Laryngeal cancer, and Nasopharyngeal cancer^69^. A major limitation of our study is that we have only used OSCC (SCC-25) and Hypopharyngeal cancer (FaDu) cell lines for this study. Hence, there is a need to study the role of LINC01518 in other subtypes of HNSCC to ensure broader validation of our findings. Moreover, our results warrant further preclinical studies on LINC01518 using low-passage patient-derived cells and animal experiments to firmly establish its role as a therapeutic target for HNSCC.

## Electronic supplementary material

Below is the link to the electronic supplementary material.

Supplementary Material

## Data Availability

The data generated or analyzed included in this article are available from the corresponding author upon reasonable request.
