# Supplementary material for: LINC01518 functions as an oncogene in head and neck squamous cell carcinoma (HNSCC) by modulating miR-1-3p/Slug and miR-216b-5p/GRP78 axis
Source: Sci Rep. 2025 Jul 2;15:22589. doi: 10.1038/s41598-025-06934-6 (PMC12217592; doi:10.1038/s41598-025-06934-6)
Supplement: Supplementary file 1 — Supplementary Material [file 41598_2025_6934_MOESM1_ESM.docx]

**Supplementary File**

**LINC01518 functions as an oncogene in Head and Neck Squamous Cell Carcinoma (HNSCC) by modulating *miR-1-3p*/Slug and *miR-216b-5p*/GRP78 axis.**

Swati^1^, Shraddha Tripathi^1^, Bakhya Shree^1^, Suryansh Sengar^1^, Amit Mishra^2^, Vivek Sharma^1^*

^1^Department of Biological Sciences, Birla Institute of Technology and Science, Pilani, Hyderabad Campus, Jawahar Nagar, Kapra Mandal, Medchal District, Telangana 500078, India

^2^Cellular and Molecular Neurobiology Unit, Indian Institute of Technology Jodhpur, Jodhpur, Rajasthan, India.

* Corresponding author: Vivek Sharma, Department of Biological Sciences, Birla Institute of Technology and Science, Pilani, Hyderabad Campus, Jawahar Nagar, Kapra Mandal, Medchal District, Telangana 500078, India

Email: [viveksharma@hyderabad.bits-pilani.ac.in](about:blank)

**Supplementary Methods**

**Bioinformatic Analysis**

LINC01518 expression analysis from 500 primary HNSCC tumor cases and 44 controls from the TCGA dataset was analyzed using the UALCAN database ^1^. Target genes for *miRNAs* were predicted using the Targetscan or miRWalk database ^2,3^. Expression analysis of *miR-1-3p*, *miR-216b-5p*, Slug, and GRP78 in HNSCC TCGA samples was done using the UALCAN database ^1^. Kaplan-Meier (KM) survival analysis for Slug and GRP78 was performed using the Protein Atlas database ^4^.

**Chromatin Immunoprecipitation (ChIP)**

Chromatin immunoprecipitation (ChIP) was performed as described previously ^5,6^. Briefly, control or TGF-β-treated SCC-25 cells were fixed with 1% formaldehyde for 8 min at room temperature, followed by quenching with 0.125 M glycine for 5 min. Cells were washed twice with ice-cold phosphate-buffered saline, harvested by scraping, pelleted, and resuspended in 250 μl of ChIP lysis buffer (50 mM Tris–HCl [pH 8.1], 0.9% SDS, 10 mM EDTA, protease inhibitor), and samples were incubated on ice for 1 h. Bioruptor Plus (Diagenode) is used to sonicate the samples using 30s on and 30s off for 24 cycles. After sonication, samples were centrifuged at 14,000 rpm at 4°C for 20 min. The supernatant is diluted by 5-folds in ChIP dilution buffer (167 mM NaCl, 16.7 mM Tris–HCl pH 8.1, 1.2 mM EDTA, 1.1% Triton X-100, protease inhibitors), 30 μg chromatin samples were used for IP, and 5% of the sample was taken as input. Samples were incubated with SMAD2/3 (1:200, CST, #8685S) or anti-rabbit IgG (CST, #2729S) antibody for overnight at 4°C. The following day, 50 μl per sample Dynabeads and protein G (Invitrogen, #10004D) were added to the IP tubes and incubated for 2 h at 4°C. The beads were washed with low-salt buffer (0.1% SDS, 1% Triton X-100, 2 mM EDTA, 20 mM Tris–HCl pH 8.1, 150 mM NaCl), high-salt buffer (0.1% SDS, 1% Triton X-100, 2 mM EDTA, 20 mM Tris HCl pH 8.1, 500 mM NaCl), LiCl buffer (0.25 M LiCl, 1% NP-40, 1% deoxycholate, 1 mM EDTA, 10 mM Tris–HCl pH 8.1), and TE buffer (pH 8.0). After this, the samples were reverse cross-linked using decrosslinking buffer (222 mM NaCl, 50 mM Tris, 10 mM EDTA, 0.025% SDS), containing 5 μl proteinase K (NEB, #P8107S, 800 U/ml) per sample with an overnight incubation at 65°C. Genomic DNA was extracted using a DNA purification kit (Zymo, #D3020) as per the manufacturer's instruction, and expression of lncRNA-LINC01518 in immunoprecipitated samples was measured using qRT-PCR.

**Plasmid construction**

The full-length human LINC01518, Slug-3’UTR, and GRP78-3’UTR were cloned into the pmirGLO vector (Promega, #E1330) using NheI and SacI restriction sites. The primers used for cloning are mentioned in Supplementary Table III. The SlugMyc_pcDNA3 construct is a gift from Paul Wade (Addgene, #31698). The pcDNA3.1(+)-GRP78/BiP construct is a gift from Richard C. Austin (Addgene, #32701).

**Cell Proliferation Assay**

Colorimetric cell proliferation assay was performed using the WST-1 reagent (Roche, 05015944001) at indicated times as described previously ^5,6^.

**Colony Formation Assay**

HNSCC cells were seeded in a 96-well plate and transfected with ASO-NS or ASO-1/ASO-2 targeting LINC01518. After 24 h, cells were trypsinized, and around 500 cells were re-seeded in a 6-well plate and incubated at 37°C for ten days, with media replacement every four days. After ten days, cells were fixed with 4% paraformaldehyde (PFA) and stained with crystal violet (Sigma, #V5265).

**Caspase 3/7 Assay**

A caspase-3/7 luminometric assay kit (Promega, #G8090) was used to determine the enzymatic activity of caspase-3/7 in HNSCC cells transfected with ASO-NS or ASO-1/ASO-2 as described previously ^5,6^.

**Invasion Assay**

HNSCC cells were transfected with ASO-NS or ASO-1/ASO-2. A day after transfection, cells were resuspended in 120 μl serum-free medium and seeded into the upper chamber of transwell inserts (Corning, #3422) precoated with Matrigel solution (Corning, #356234). The bottom chamber contained media with 20% FBS. After 48 h, the cells on the upper chamber of transwell inserts were scraped off by a cotton swab, and the invaded cells were fixed with 4% PFA and stained with a crystal violet solution. Transwell inserts with stained cells were examined under the microscope, and the percentage of cells invaded was evaluated using ImageJ as described previously ^5,6^.

**Migration Assay**

HNSCC cells were transfected with either ASO-NS or ASO-1/ASO-2 in a 12-well plate. Forty-eight hours later, when cells reached ~95 % confluency, a scratch was made using a 20ul pipette tip, and cells were washed with 1X-PBS and cultured in low serum-containing media (0.5% FBS). The images were captured after 0 h, 24 h, and 48 h of creating the scratch using a light microscope (Magnus INVI microscope). To calculate the percentage of migrated cells, lines were drawn along the leading edges of the cells, and the gap distances of migrating cells from three different areas for each scratch were measured using ImageJ ^5,6^. The average distance from three different areas of migrated cells at 24 h or 48 h was subtracted from the average distance of migrated cells at 0 h. The percentage migration was calculated for 24 h and 48 h time points by normalizing the migrated distance of ASO-1/2 transfected cells to the migrated distance of ASO-NS transfected cells.

**Supplementary Figures**

**Figure S1. LINC01518 knockdown in HNSCC cells impairs colony formation, invasion, and migration.**

**
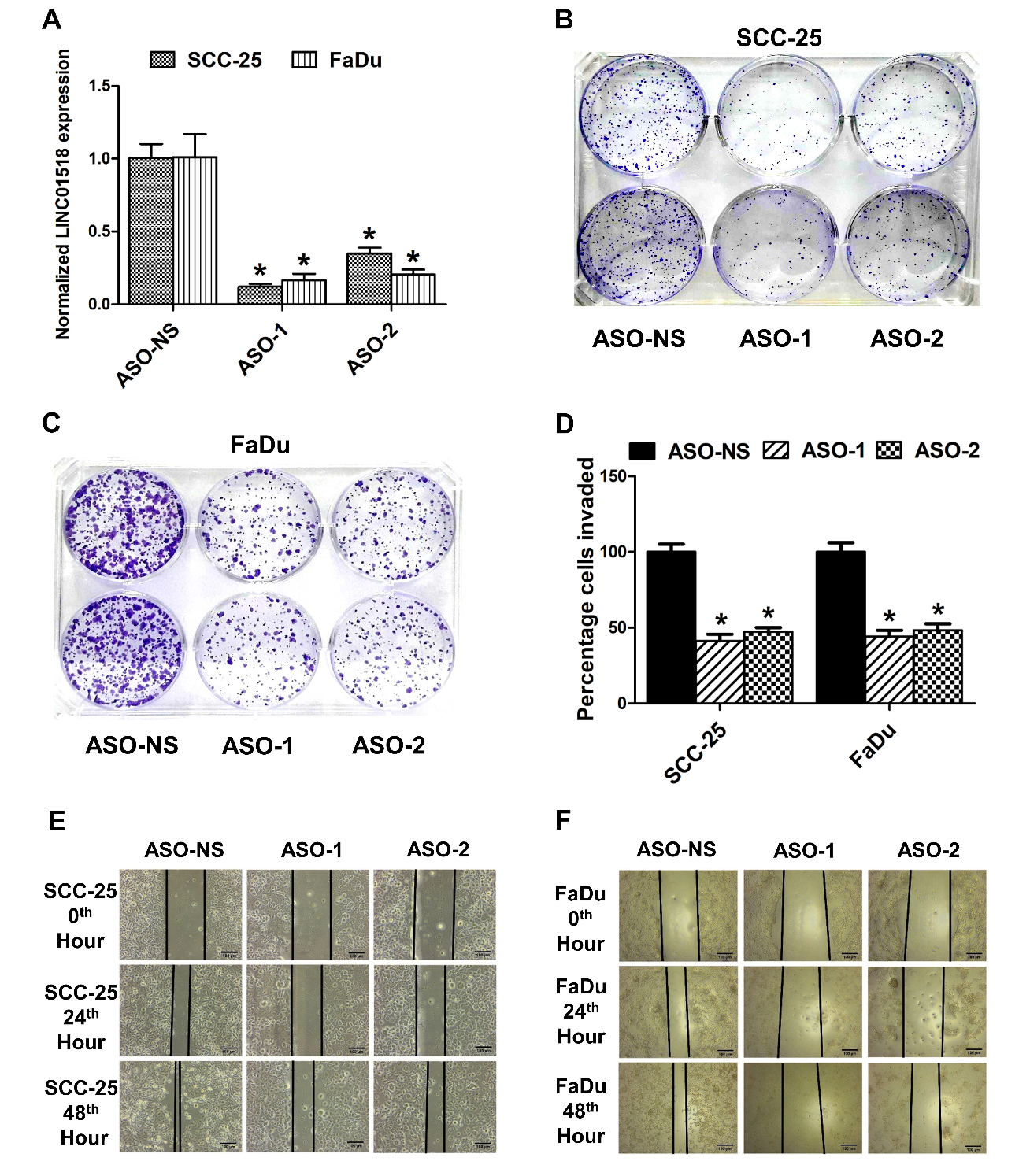
**

1. Efficiency of ASO-mediated knockdown of LINC01518 in HNSCC cells. LINC01518 expression as detected by qRT-PCR.
2. Representative image of colony formation of SCC-25 cells after LINC01518 knockdown.
3. Representative image of colony formation of FaDu cells after LINC01518 knockdown.
4. Quantification of invasion shown in Fig 2E.
5. Representative images of migration upon LINC01518 knockdown in SCC-25 cells.
6. Representative images of migration upon LINC01518 knockdown in FaDu cells.

**Data information: Error bars represent the mean ± SEM from three independent experiments. *Significant change compared to ASO-NS (p < 0.05). Statistical comparisons were made using the Student’s t-test.**

**Figure S2. Effect of LINC01518 knockdown on genes associated with TGF-β pathway.**


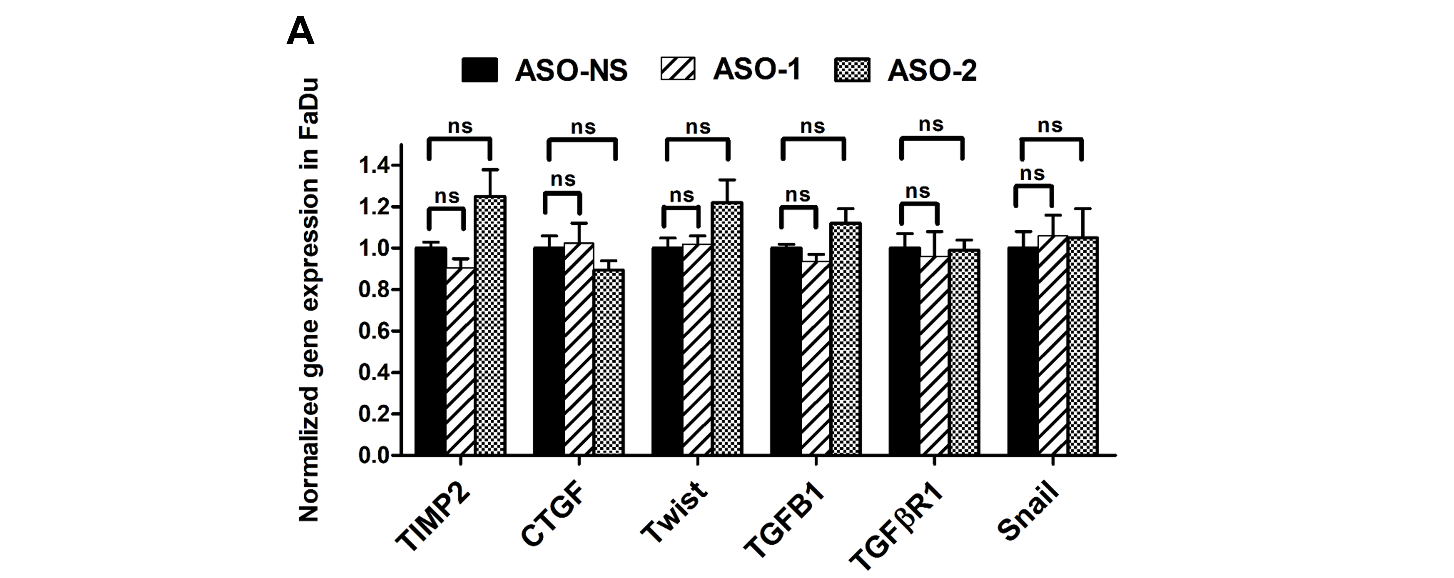


Expression of many TGF-β target genes does not change upon LINC01518 knockdown. Gene expression of TGF-β target genes upon LINC01518 knockdown measured using qRT–PCR in FaDu cells.

**Data information: Error bars represent the mean ± SEM from three independent experiments. ns: non-significant change compared to ASO-NS (p>0.05). Statistical comparisons were made using the Student's t-test.**

P.T.O.

**Figure S3. LINC01518 sponges *miR-1-3p* to promote Slug expression in HNSCC.**

**
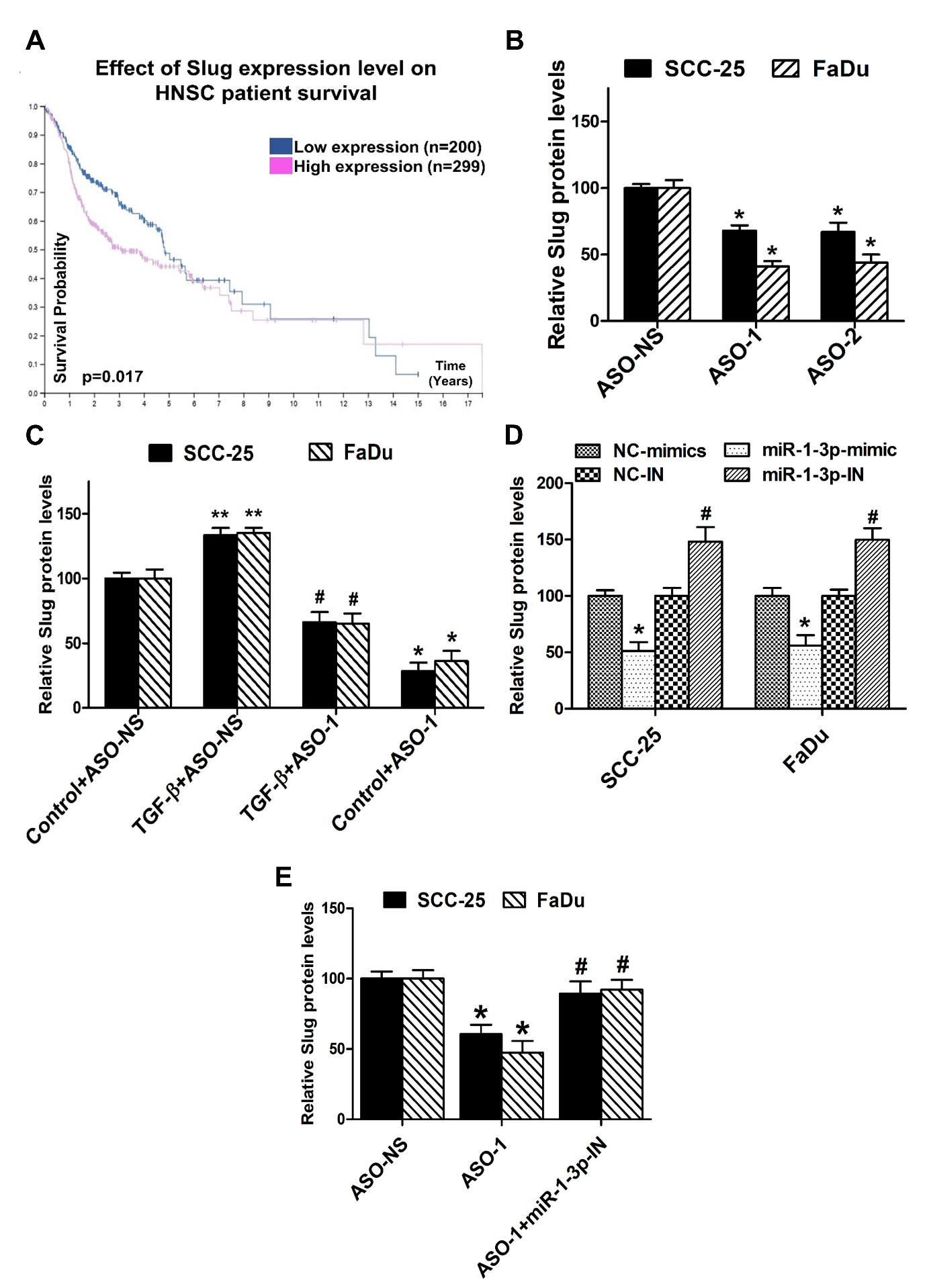
**

1. KM survival analysis of HNSCC patients shows that high Slug expression is correlated with poor overall survival in HNSCC patients. The pink line represents the high Slug expression group, and the blue line represents the low Slug expression group. The survival analysis was done using the protein atlas database.
2. Quantification of western blots of Slug shown in Fig. 5B.
3. Quantification of western blots of Slug shown in Fig. 5C.
4. Quantification of western blots of Slug shown in Fig. 5G.
5. Quantification of rescue blots of Slug shown in Fig. 5H after LINC01518 knockdown and *miR-1-3p* inhibition in HNSCC cells.

**Data information: Error bars represent the mean ± SEM from three independent experiments. (For B) *Significant change as compared to ASO-NS (p < 0.05). (For C) **Significant change compared to Control+ASO-NS cells (p < 0.05). ^#^Significant change as compared to TGF-β+ASO-NS cells (p < 0.05). *Significant change as compared to Control+ASO-NS cells (p < 0.05). (For D) *Significant change as compared to NC-mimic (p < 0.05). ^#^Significant change as compared to NC-Inhibitor (p < 0.05). (For E) *Significant change as compared to ASO-NS (p < 0.05). ^#^Significant change as compared to ASO-1 (p < 0.05). Statistical comparisons were made using the Student's t-test.**

P.T.O.

**Figure S4. LINC01518 sponges *miR-216b-5p* to promote GRP78 expression in HNSCC.**

**
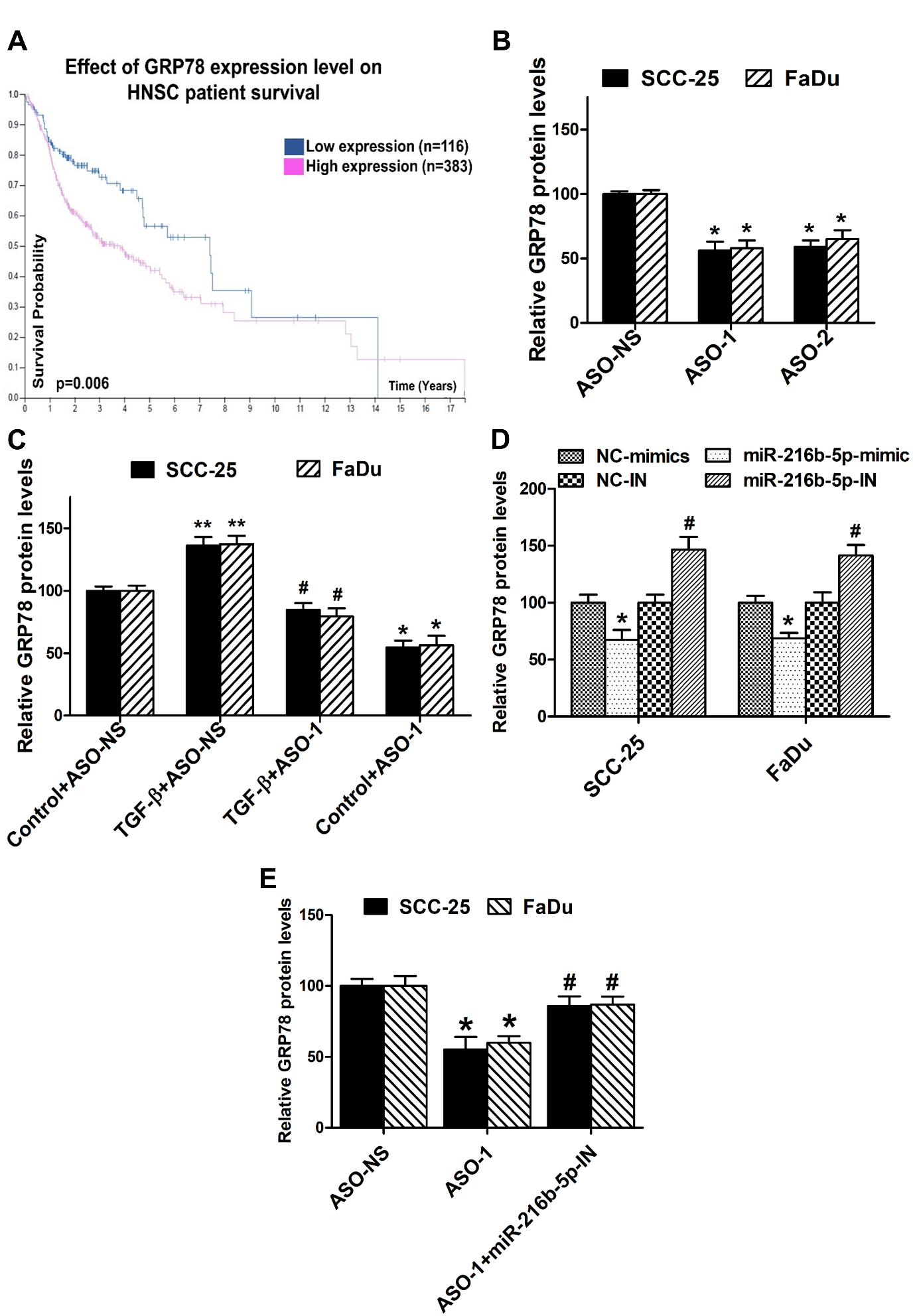
**

1. KM survival analysis of HNSCC patients shows that high GRP78 expression is correlated with poor overall survival in HNSCC patients. The pink line represents the high GRP78 expression group, and the blue line represents the low GRP78 expression group. The survival analysis was done using the protein atlas database.
2. Quantification of western blots of GRP78 shown in Fig. 6B.
3. Quantification of western blots of GRP78 shown in Fig. 6C.
4. Quantification of western blots of GRP78 shown in Fig. 6G.
5. Quantification of rescue blots of GRP78 shown in Fig. 6H after LINC01518 knockdown and *miR-216b-5p* inhibition in HNSCC cells.

**Data information: Error bars represent the mean ± SEM from three independent experiments. (For B) *Significant change as compared to ASO-NS (p < 0.05). (For C) **Significant change as compared to Control+ASO-NS cells (p < 0.05). ^#^Significant change as compared to TGF-β+ASO-NS cells (p < 0.05). *Significant change as compared to Control+ASO-NS cells (p < 0.05). (For D) *Significant change as compared to NC-mimic (p < 0.05). ^#^Significant change as compared to NC-Inhibitor (p < 0.05). (For E) *Significant change as compared to ASO-NS (p < 0.05). ^#^Significant change as compared to ASO-1 (p < 0.05). Statistical comparisons were made using the Student's t-test.**

**Table I: List of ASOs, miRNA mimic, and miRNA inhibitors**

| **Antisense Oligonucleotides** | **Sequence** | **Catalog no.** |
| --- | --- | --- |
| ASO-NS | AACACGTCTATACGC | Qiagen, 339515, LG00000002-DDA |
| LINC01518 ASO-1 | TACAGCGCCTTTGGTA | Qiagen, 339511, LG00781145-DDA |
| LINC01518 ASO-2 | GAGGGAGAACAACGTT | Qiagen, 339511, LG00781146-DDA |
| **miRNA mimic** | **Sequence** | **Catalog no.** |
| NC mimic | UCACCGGGUGUAAAUCAGCUUG | Qiagen, 339173, YM00479902-ADA |
| *miR-1-3p* mimic | UGGAAUGUAAAGAAGUAUGUAU | Qiagen, 339173, YM00472818-ADA |
| *miR-216b-5p* mimic | AAAUCUCUGCAGGCAAAUGUGA | Qiagen, 339173, YM00470958-ADA |
| **miRNA Inhibitors** | **Sequence** | **Catalog no.** |
| NC inhibitor | TAACACGTCTATACGCCCA | Qiagen, 339126, YI00199006-ADA |
| *miR-1-3p* inhibitor | ACATACTTCTTTACATTCCA | Qiagen, 339121, YI04100840-ADA |
| *miR-216b-5p* inhibitor | CACATTTGCCTGCAGAGATT | Qiagen, 339121, YI04101761-ADA |

**Table II: List of primers used for qRT‐PCR**

| **qRT-PCR Primers** | **Sequence (5'-3')** |
| --- | --- |
| LINC01518-FP1 | CAGTGACGGAACAGTACCAG |
| LINC01518-RP1 | TCACAAACATCCCGCTCT |
| LINC01518-FP2 | TTCTGTAGAAGGCAGTGACGG |
| LINC01518-RP2 | CTCCTCATCTTCAACGAGCG |
| Slug-FP | AGACCCTGGTTGCTTCAAGGA |
| Slug-RP | CTCAGATTTGACCTGTCTGCAAA |
| GRP78-FP | CTGTCCAGGCTGGTGTGCTCT |
| GRP78-RP | CTTGGTAGGCACCACTGTGTTC |
| Serpine1-FP | GGCTGACTTCACGAGTCTTTCA |
| Serpine1-RP | ATGCGGGCTGAGACTATGACA |
| N-cadherin-FP | TATGCCCAAGACAAAGAGACC |
| N-cadherin-RP | CAACTTCTGCTGACTCCTTCA |
| MMP2-FP | AGATGCCTGGAATGCCAT |
| MMP2-RP | GGTTCTCCAGCTTCAGGTAAT |
| Vimentin-FP | CAGCTAACCAACGACAAA |
| Vimentin-RP | CGTGGAGTTTCTTCAAAAAG |
| ZEB1-FP | ACTCTGATTCTACACCGC |
| ZEB1-RP | TGTCACATTGATAGGGCTT |
| ZEB2-FP | TGAGGATGACGGTATTGC |
| ZEB2-RP | ATCTCGTTGTTGTGCCAG |
| E-Cadherin-FP | CCTGGGACTCCACCTACAGA |
| E-Cadherin-RP | TGGATTCCAGAAACGGAGGC |
| TIMP2-FP | GGATTTTTGGGGGAGGGGAG |
| TIMP2-RP | ACCGAGCGATTGCTCAAGAA |
| CTGF-FP | GTTTGGCCCAGACCCAACTA |
| CTGF-RP | GGCTCTGCTTCTCTAGCCTG |
| Twist-FP | GTCCGCAGTCTTACGAGGAG |
| Twist-RP | TGGAGGACCTGGTAGAGGAA |
| TGFB1-FP | AGGAAAATCTGTGGCAAATCA |
| TGFB1-RP | TTGAGAGTGGTAGGGCTGCT |
| TGFβR1-FP | GCAGAGCTGTGAAGCCTTGAGA |
| TGFβR1-RP | TGCCTTCCTGTTGACTGAGTTG |
| Snail-FP | CAATCGGAAGCCTAACTA |
| Snail-RP | CAGATGAGCATTGGCAGCG |
| TBP_FP | GAGCTGTGATGTGAAGTTTCC |
| TBP-RP | TCTGGGTTTGATCATTCTGTAG |
| HPRT_FP | TGAGGATTTGGAAAGGGTGT |
| HPRT_RP | GAGCACACAGAGGGCTACAA |

**Table III: List of primers used for cloning**

| **Cloning Primers** | **Sequence (5'-3')** |
| --- | --- |
| LINC01518-pmiR-FP | TAAGCAGCTAGCCTGGAGTGGTAAGATGGCG |
| LINC01518-pmiR-RP | TGCTTAGTCGACGTGAAAAATGCTTTATTAA |
| Slug 3’ UTR-FP | ACAGAGCTCGTGACGCAATCAATGTT |
| Slug 3’ UTR-RP | GGTGTAGCTAGCTAAAAATAATACTAGCA |
| GRP78 3' UTR-FP | TAAGCAGAGCTCGGACAGGCTGGTGCTAACATA |
| GRP78 3' UTR-RP | TGCTTAGCTAGCCCCAAATGCTTTTCGGGCAG |

**Full blots for Figure 3, 5 and 6**

1. **Figure 3C – SCC-25**

**
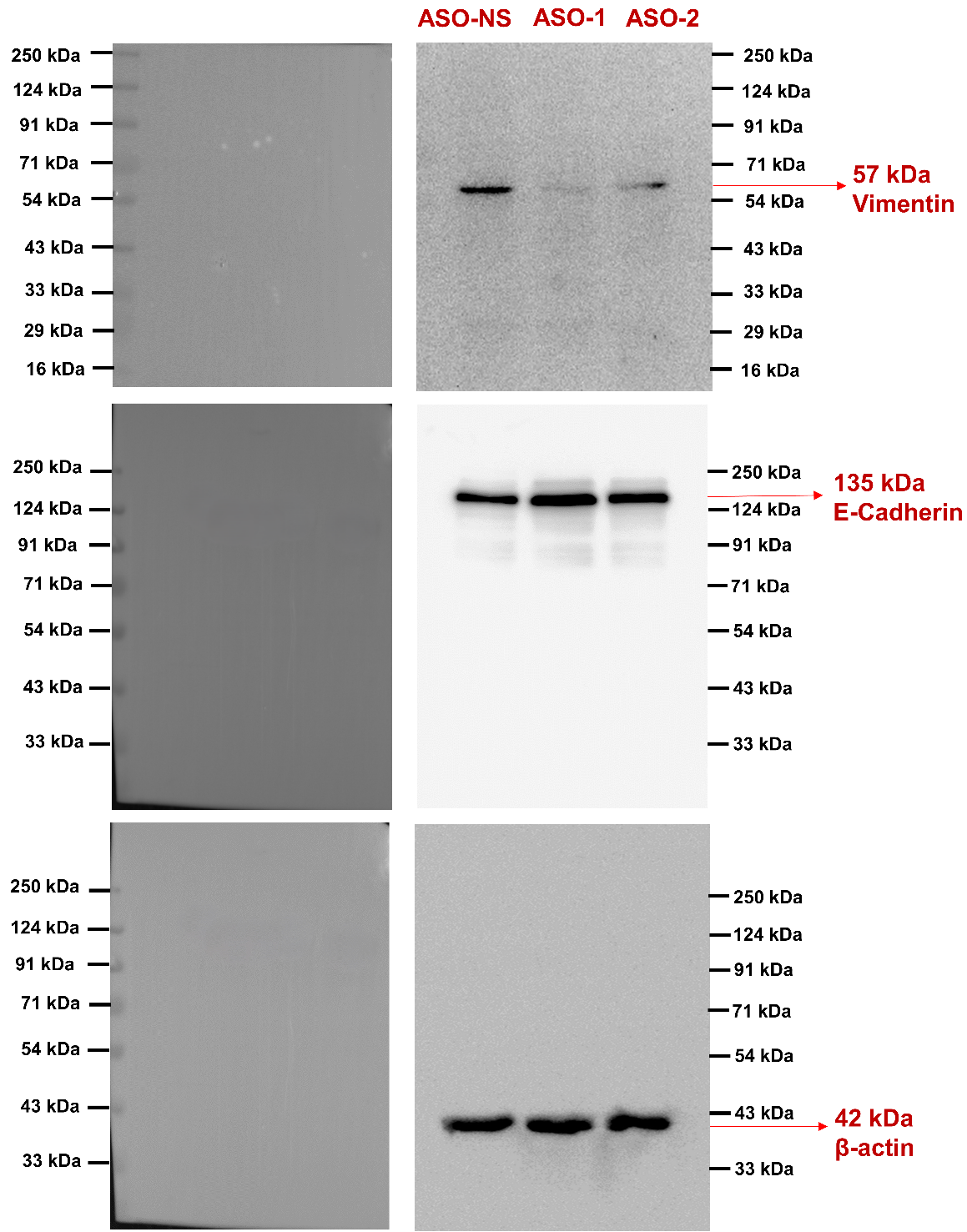
**

1. **Figure 3C – FaDu**

**
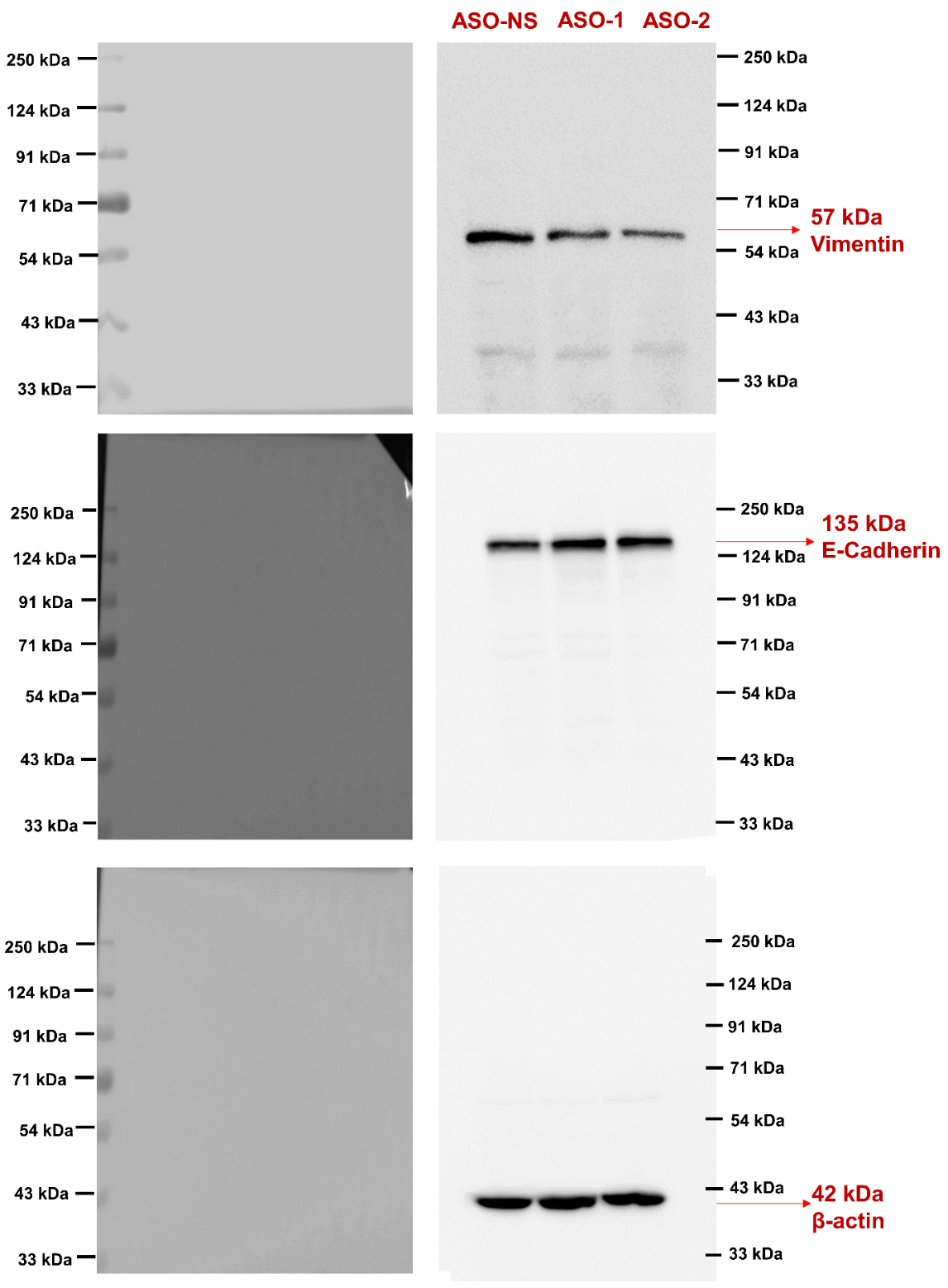
**

1. **Figure 3E – SCC-25**

**
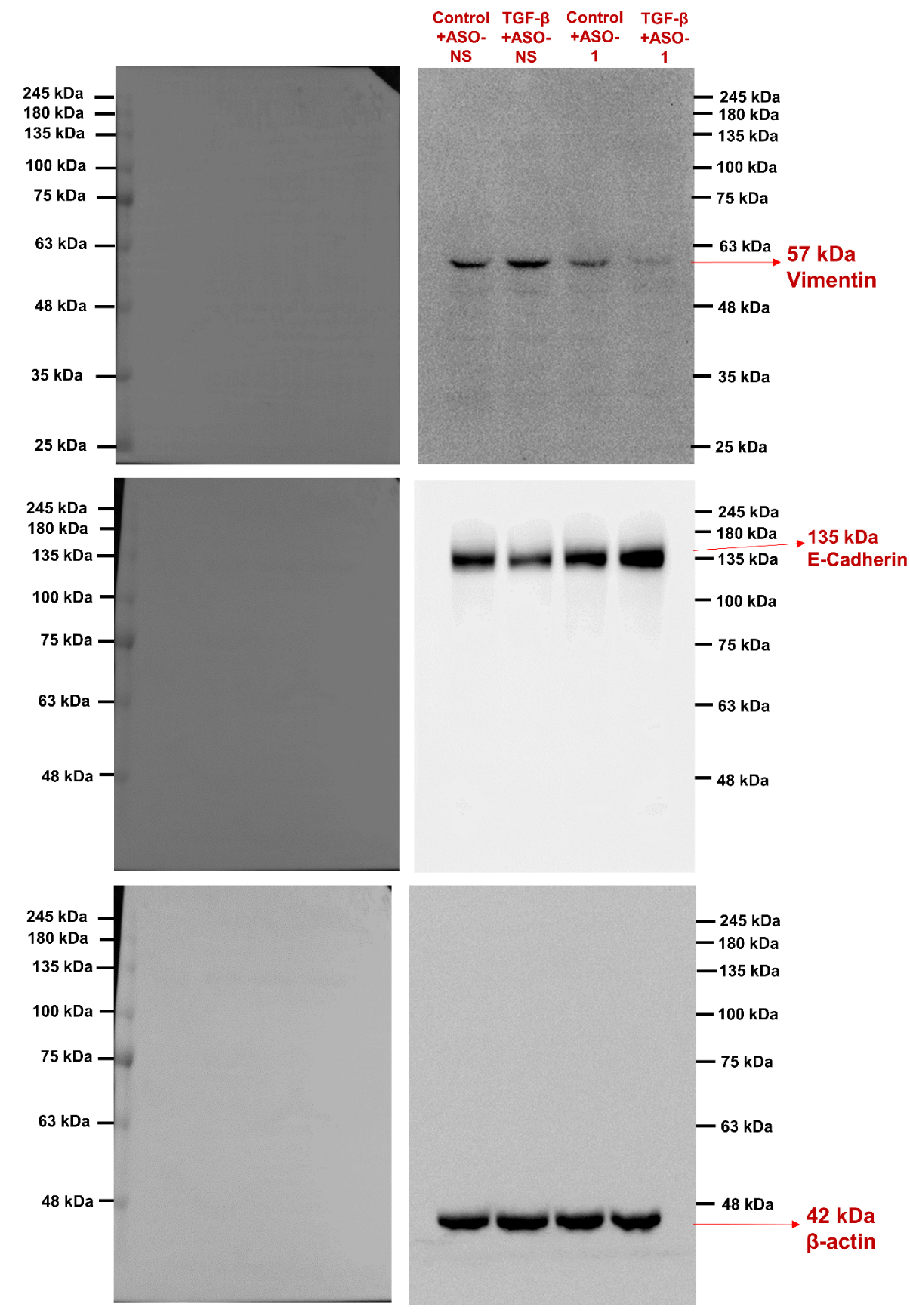
**

1. **Figure 3E – FaDu**

**
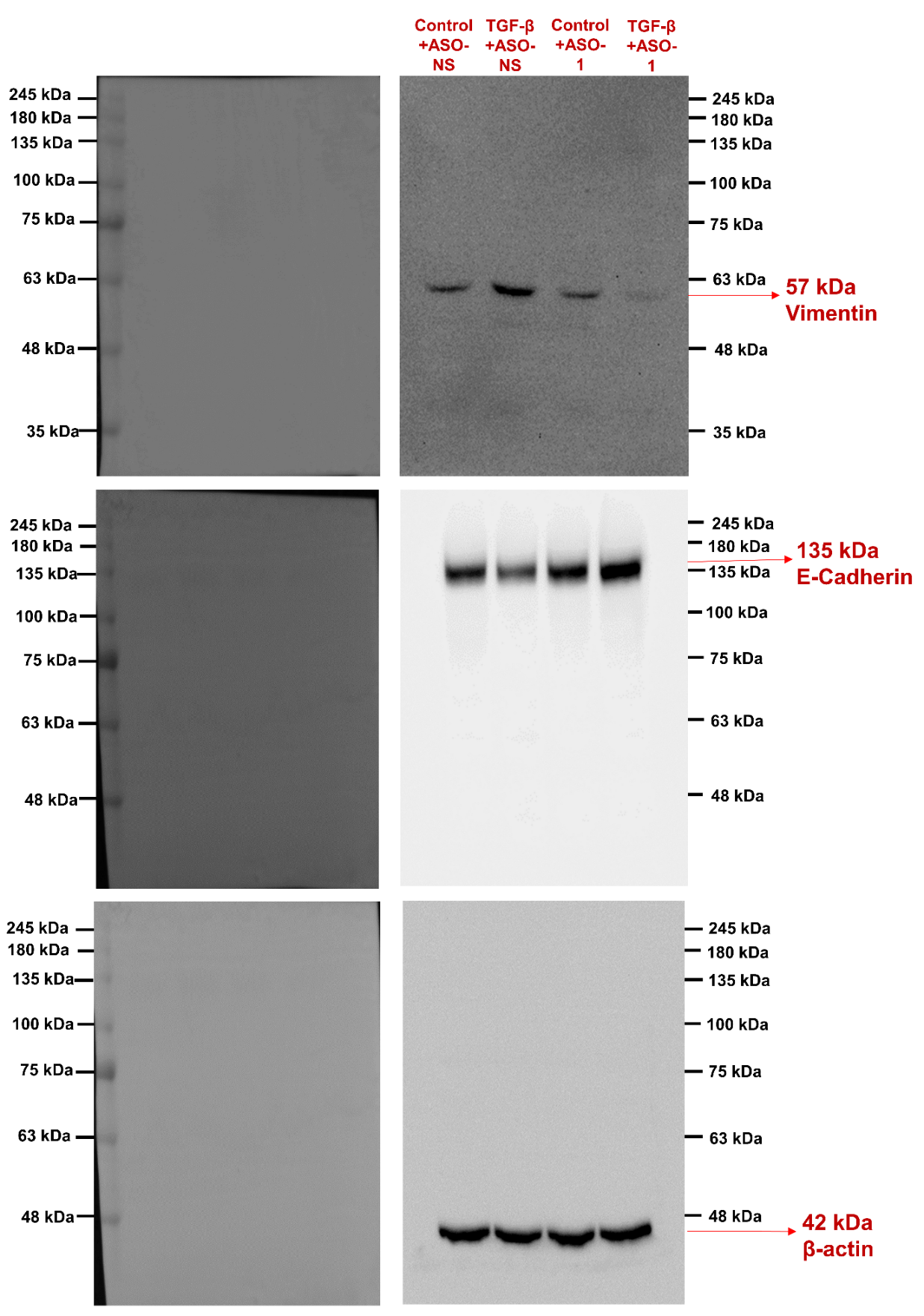
**

1. **Figure 5B– SCC-25**

**
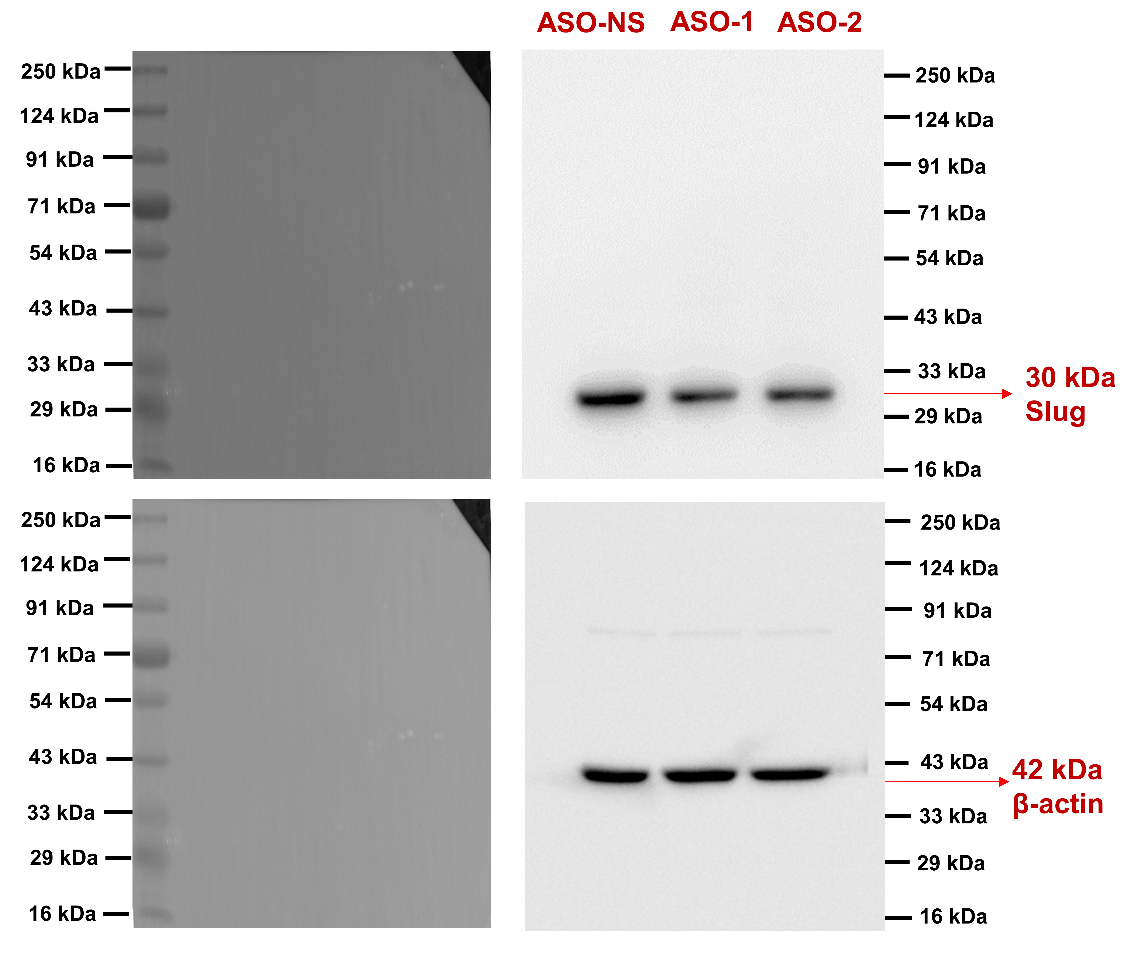
**

1. **Figure 5B– FaDu**

**
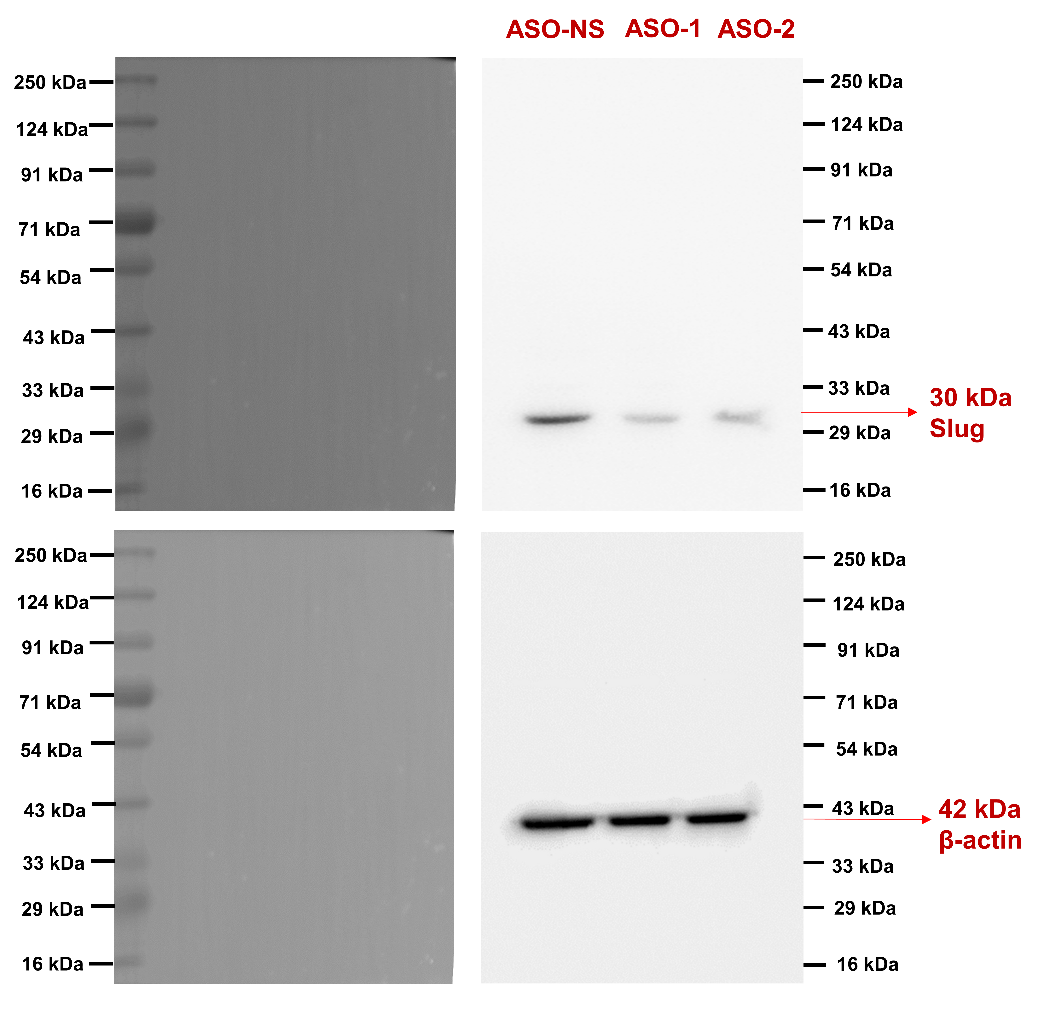
**

1. **Figure 5C– SCC-25**

**
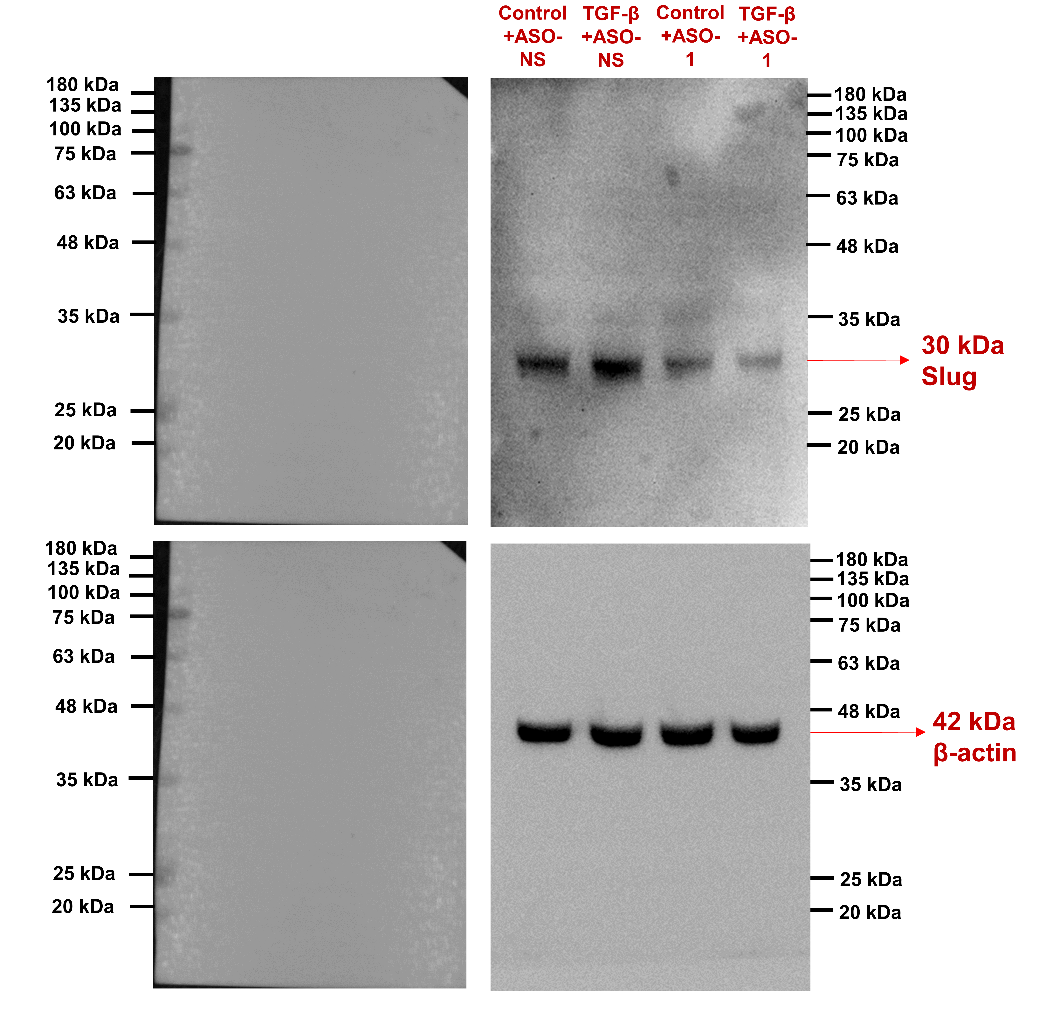
**

1. **Figure 5C– FaDu**

**
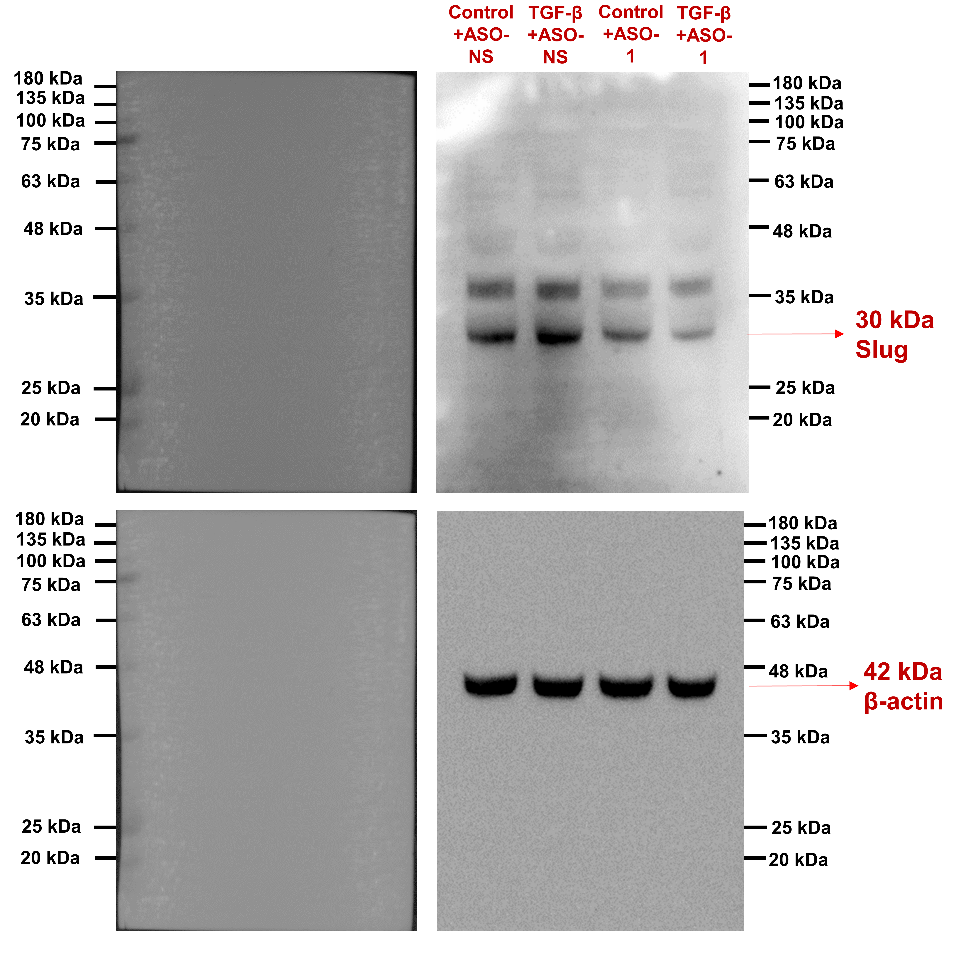
**

1. **Figure 5G– SCC-25**

**
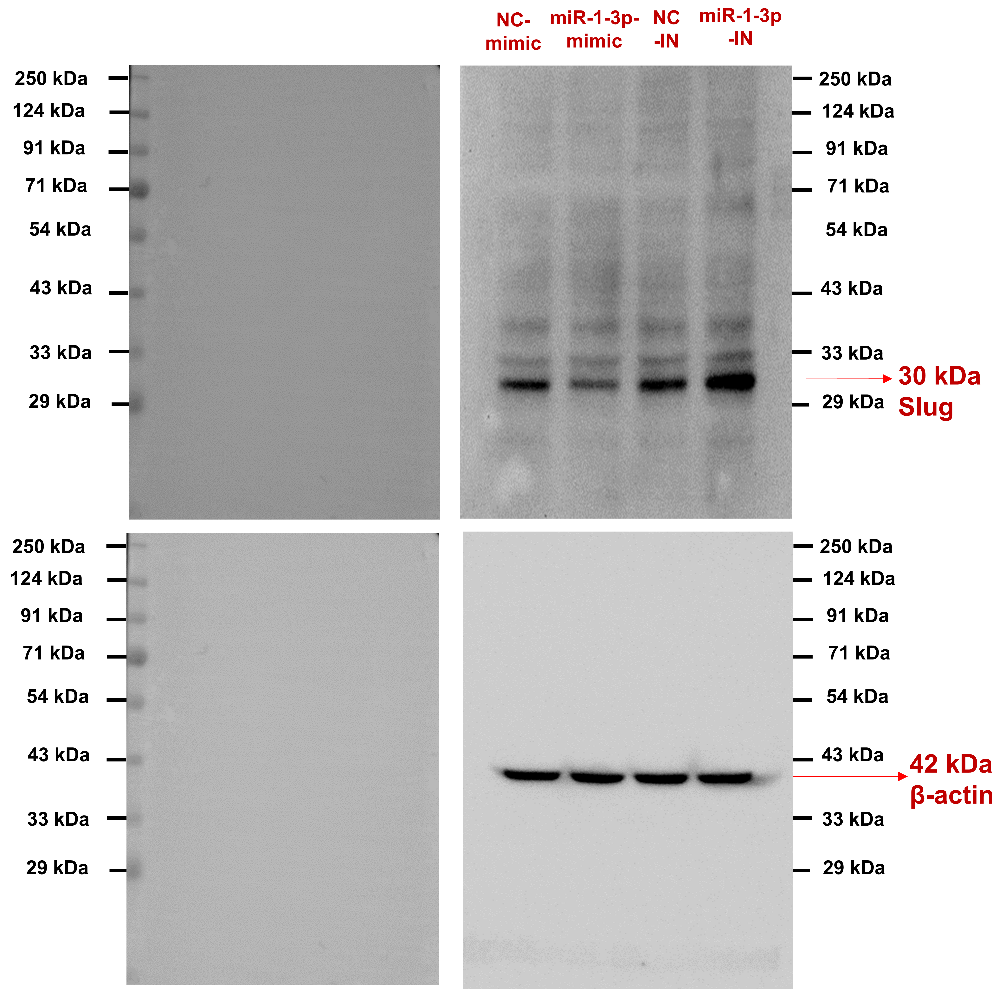
**

1. **Figure 5G– FaDu**

**
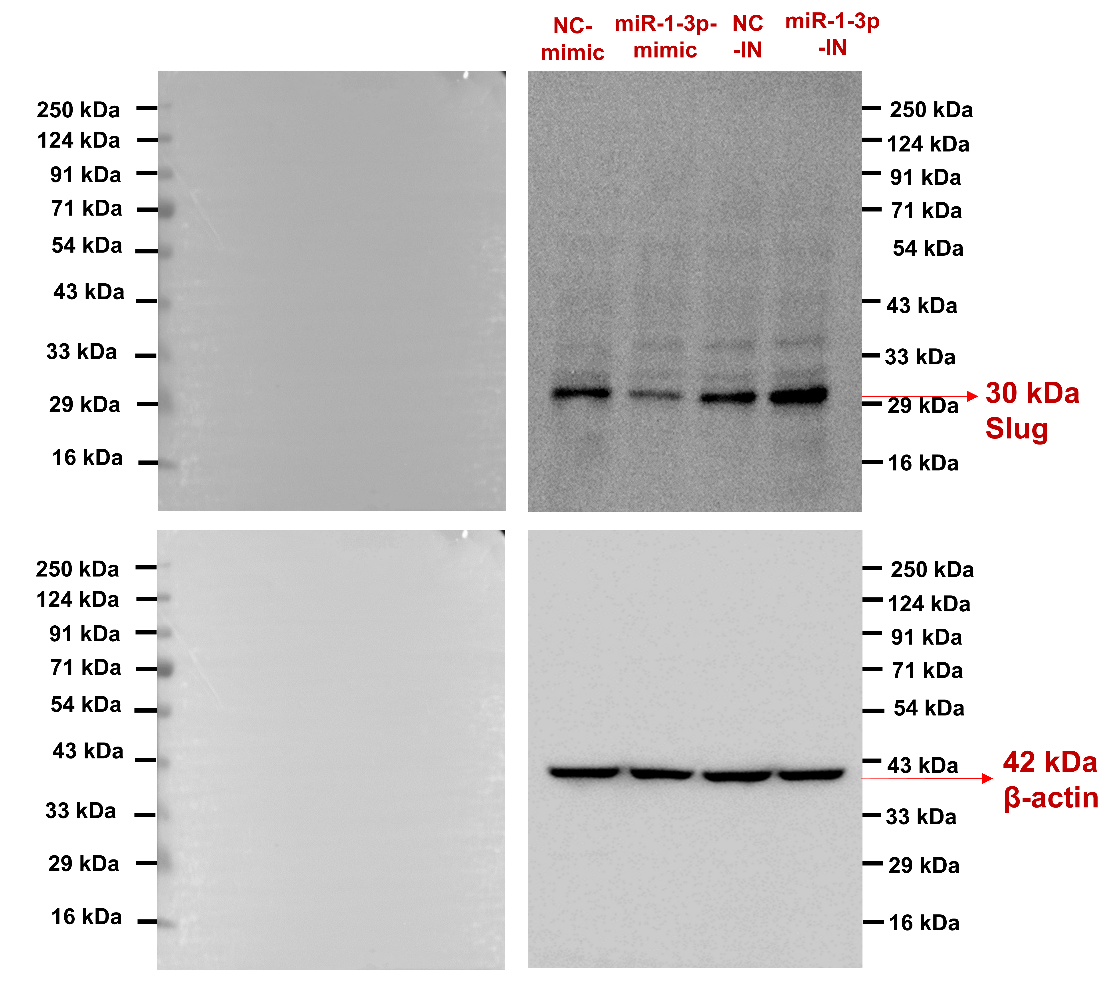
**

1. **Figure 5H– SCC-25**

**
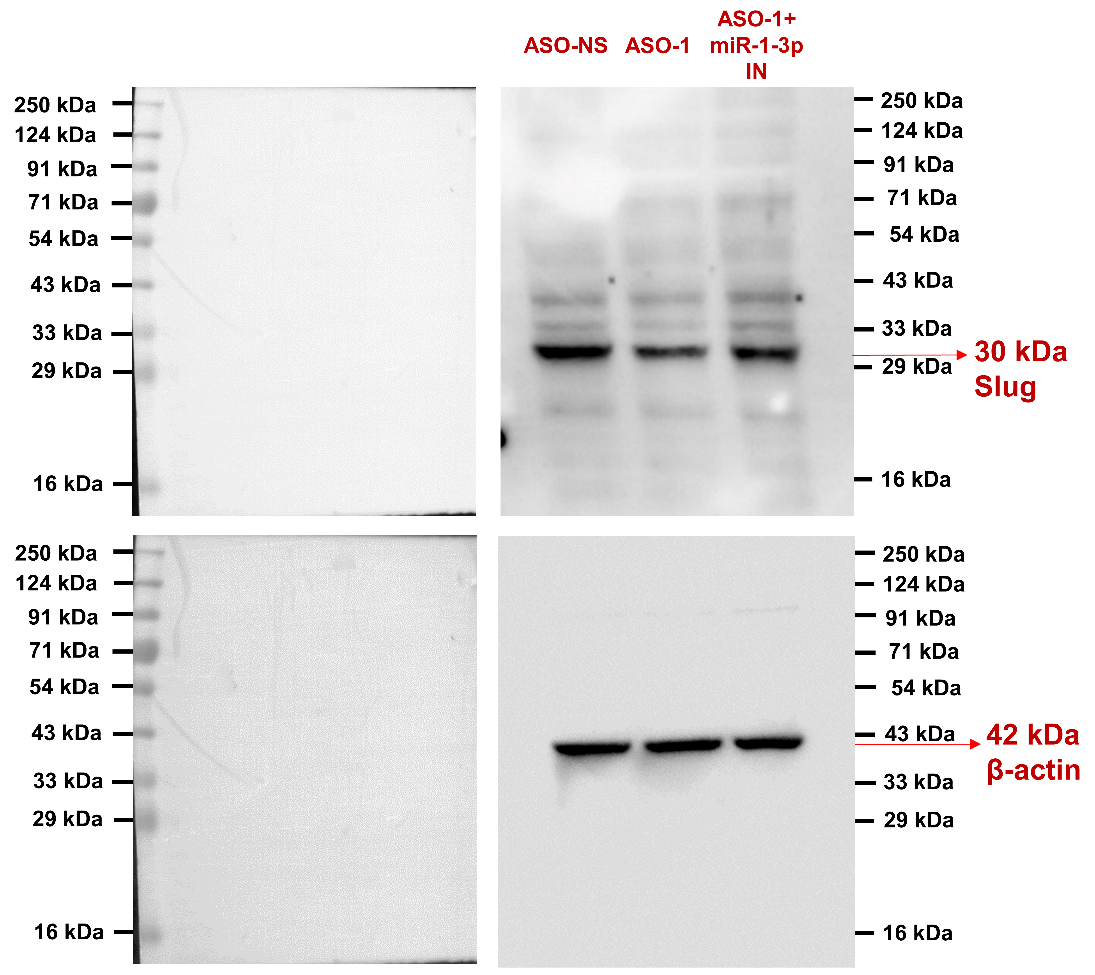
**

1. **Figure 5H– FaDu**

**
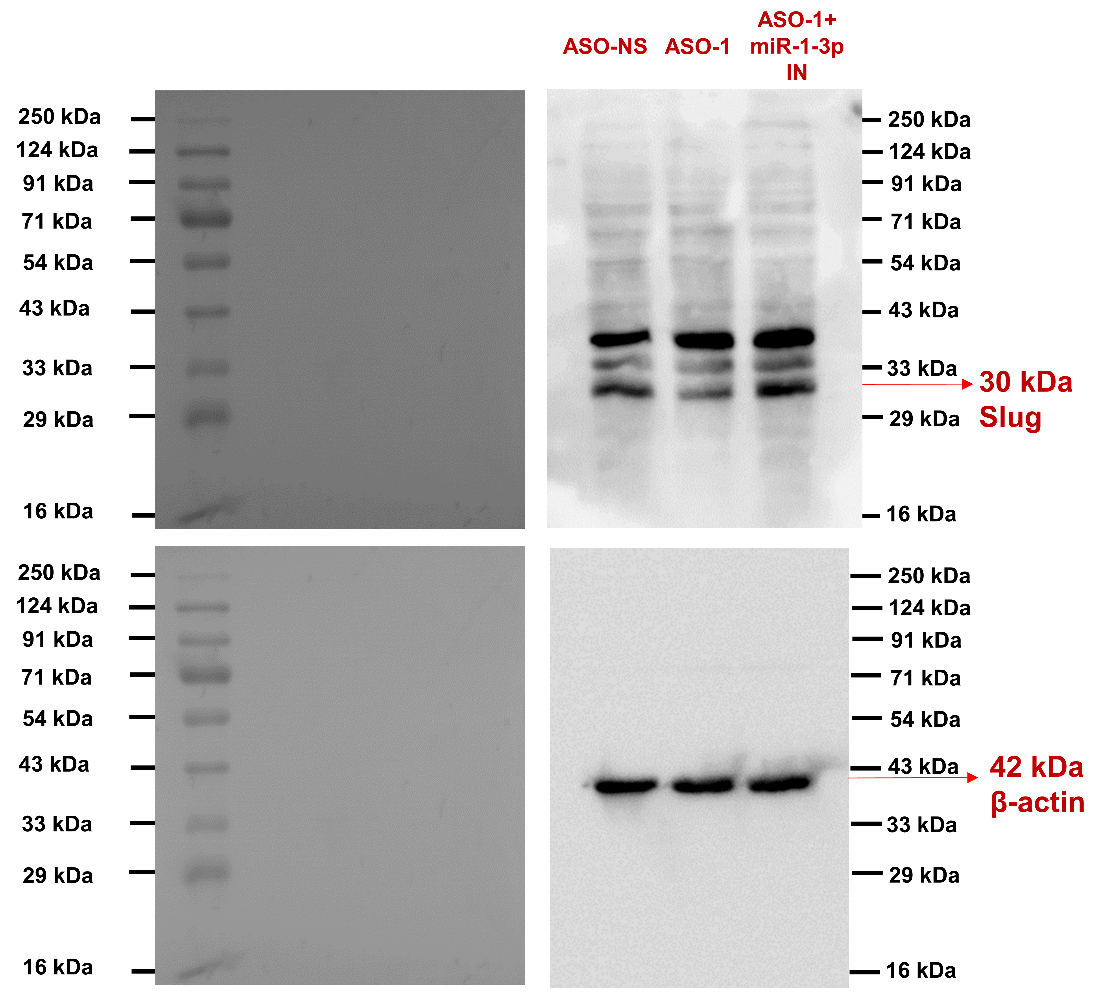
**

1. **Figure 6B– SCC-25**

**
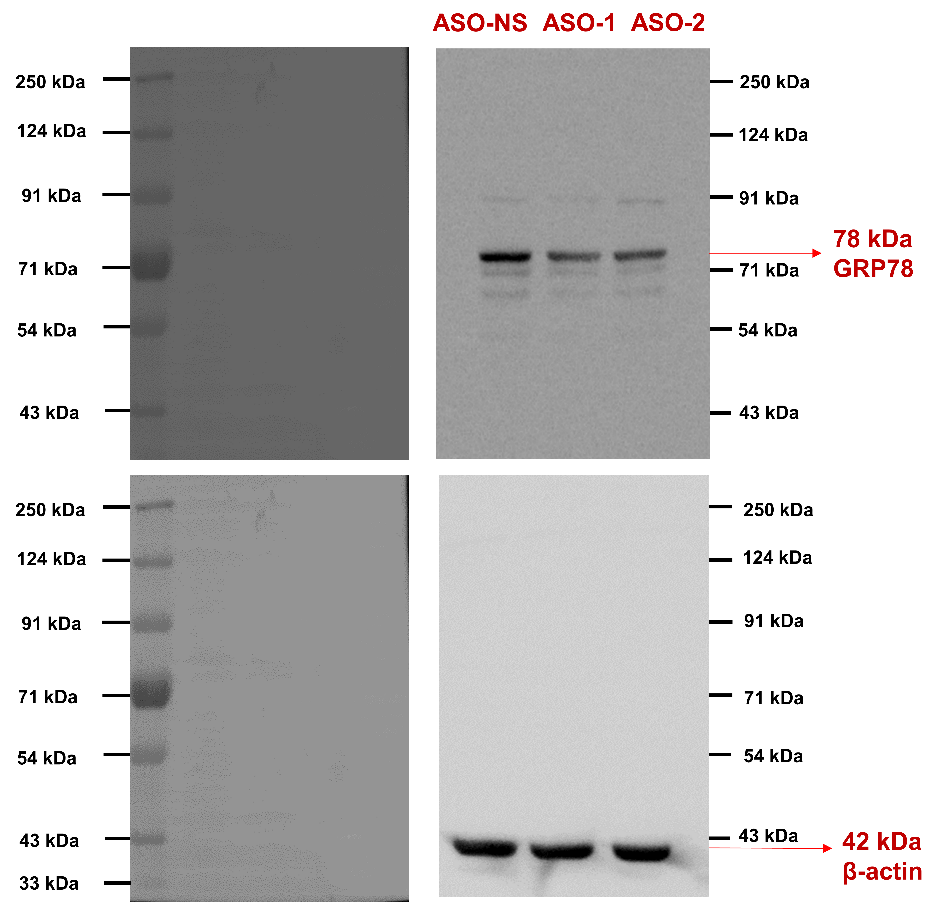
**

1. **Figure 6B– FaDu**

**
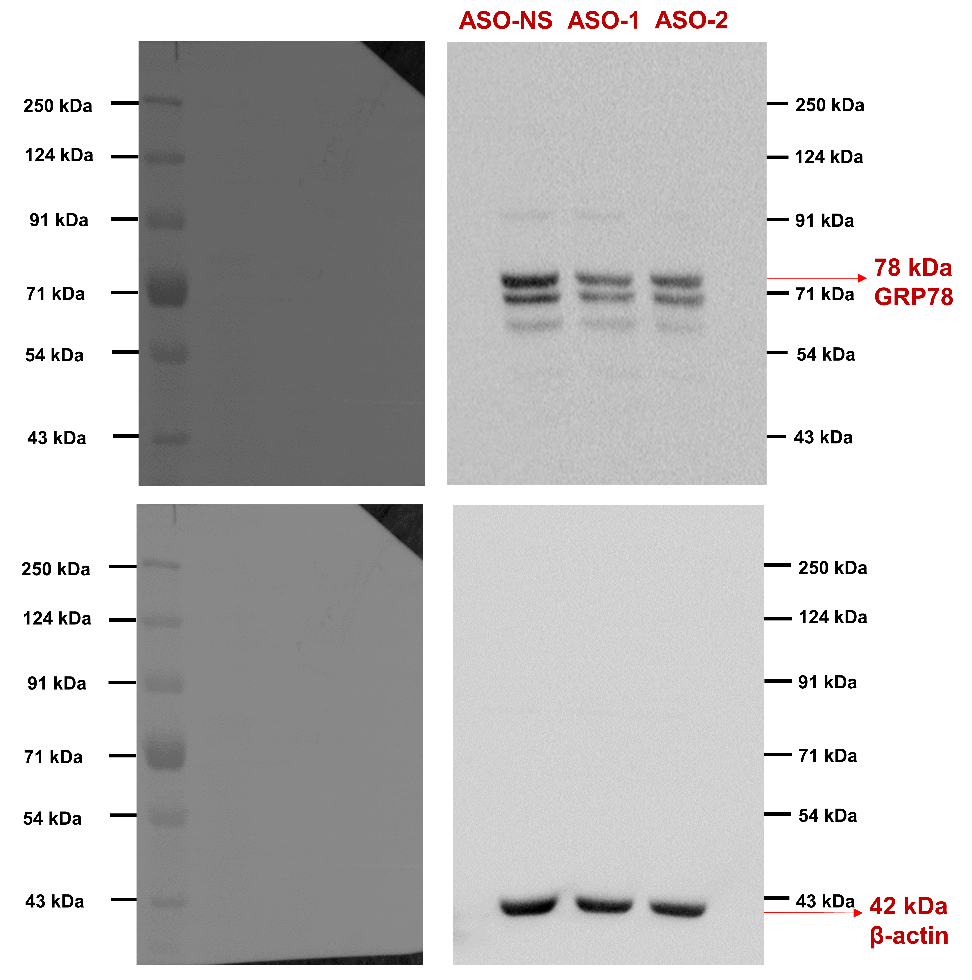
**

1. **Figure 6C– SCC-25**

**
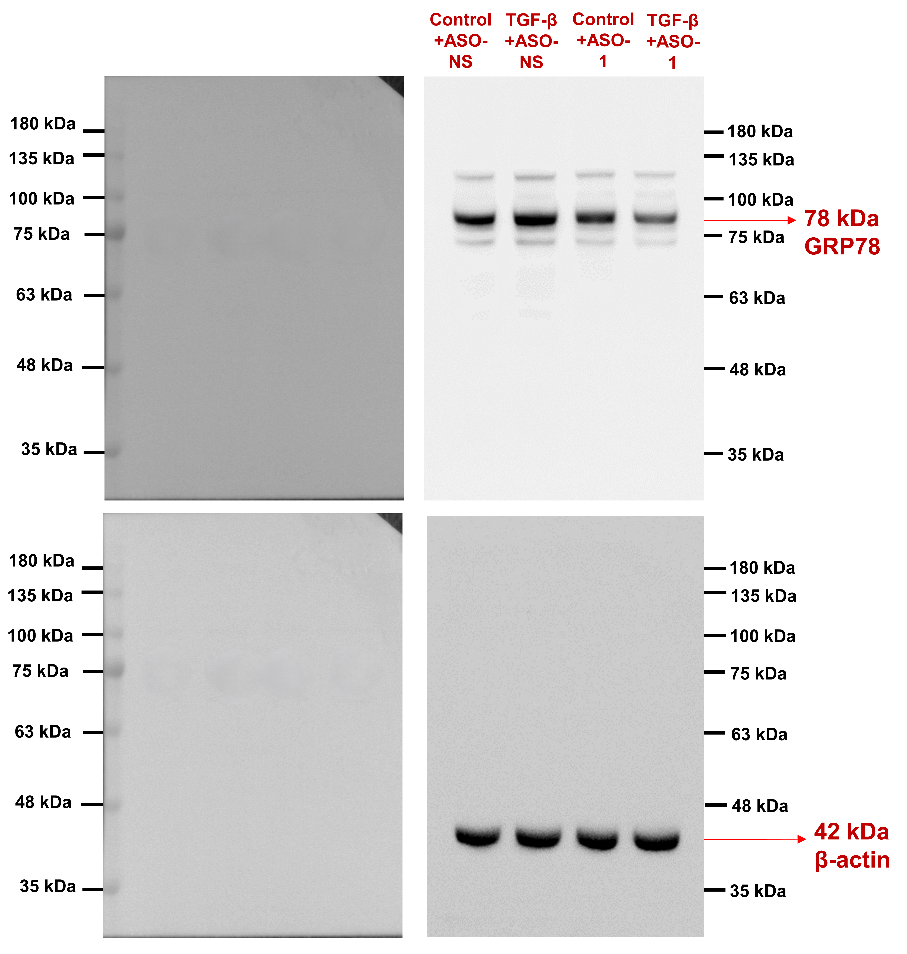
**

1. **Figure 6C– FaDu**

**
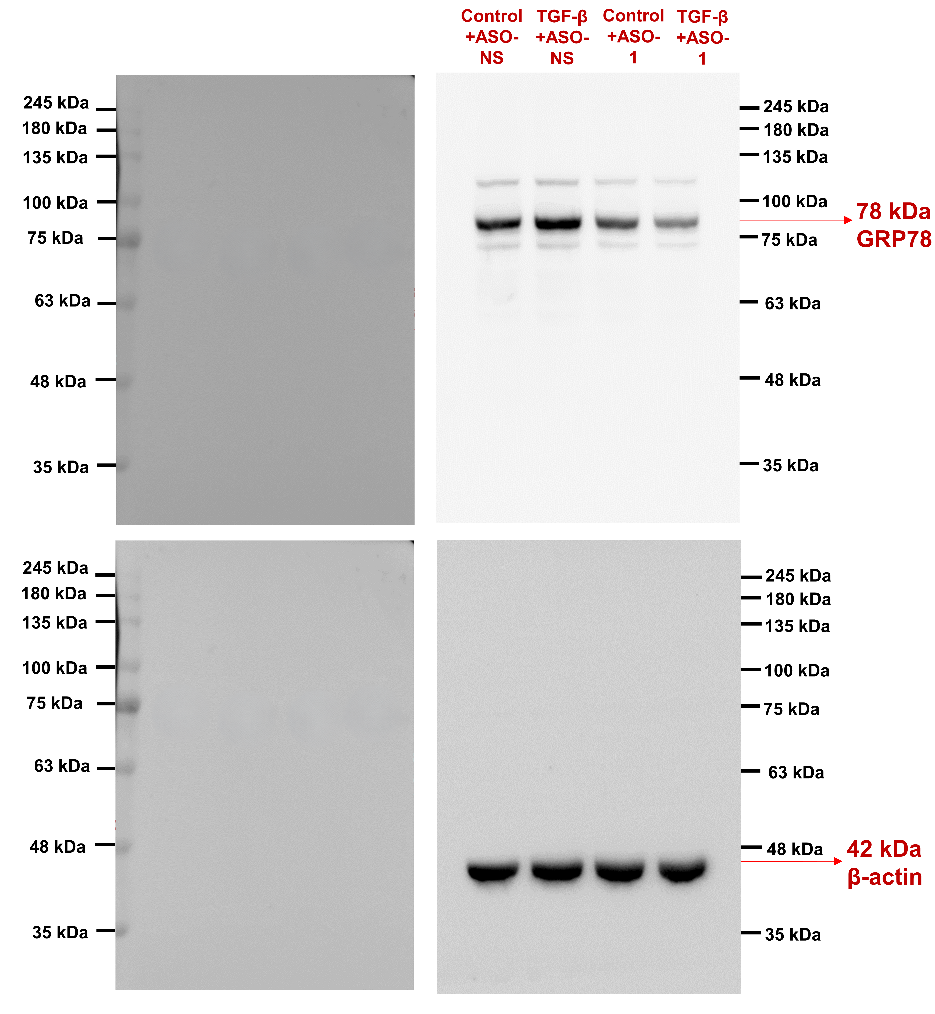
**

1. **Figure 6G– SCC-25**

**
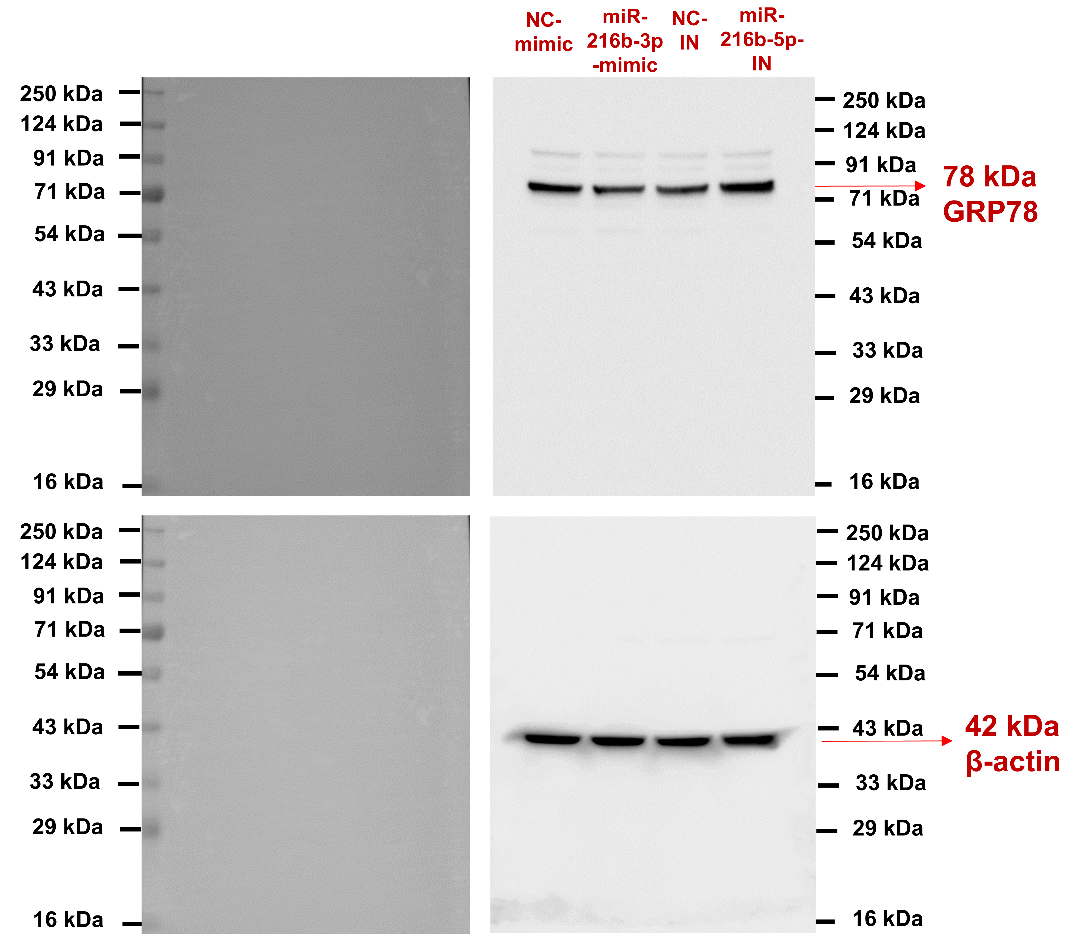
**

1. **Figure 6G– FaDu**

**
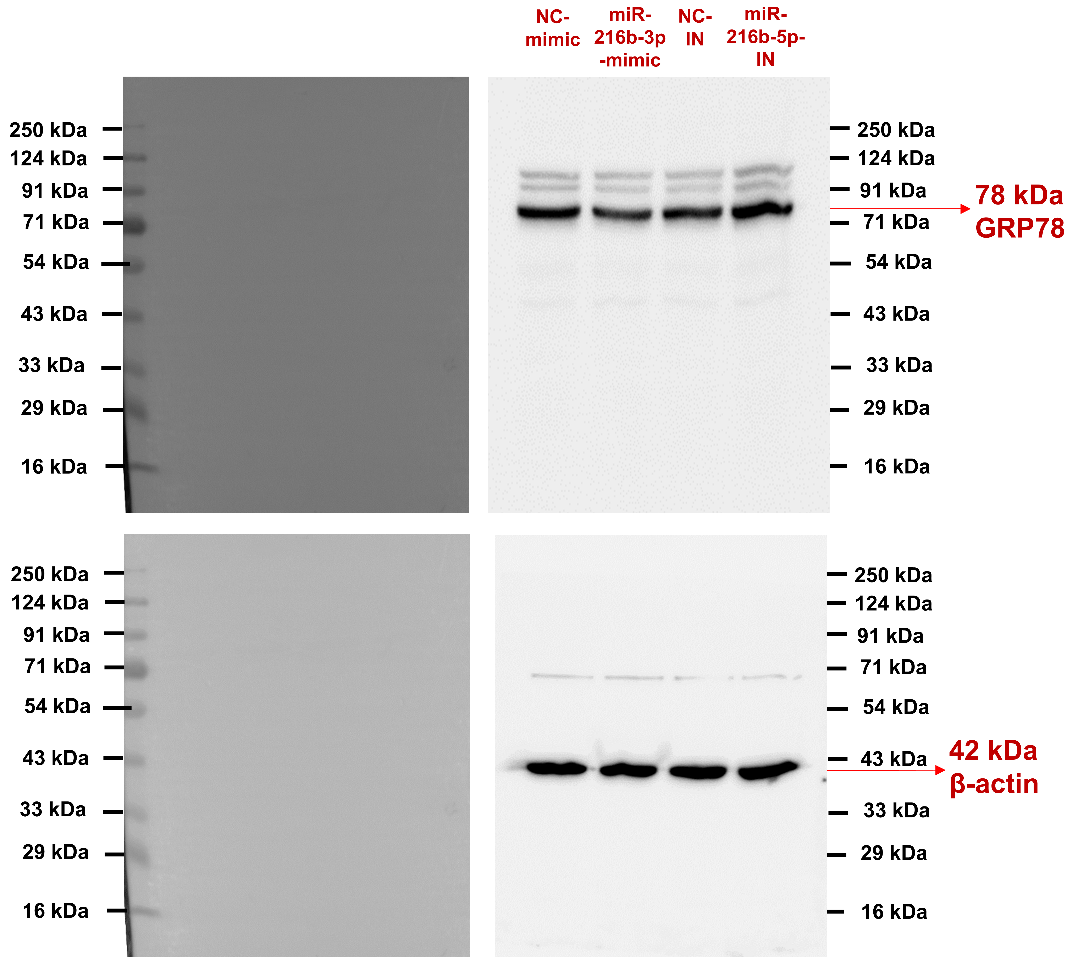
**

1. **Figure 6H– SCC-25**

**
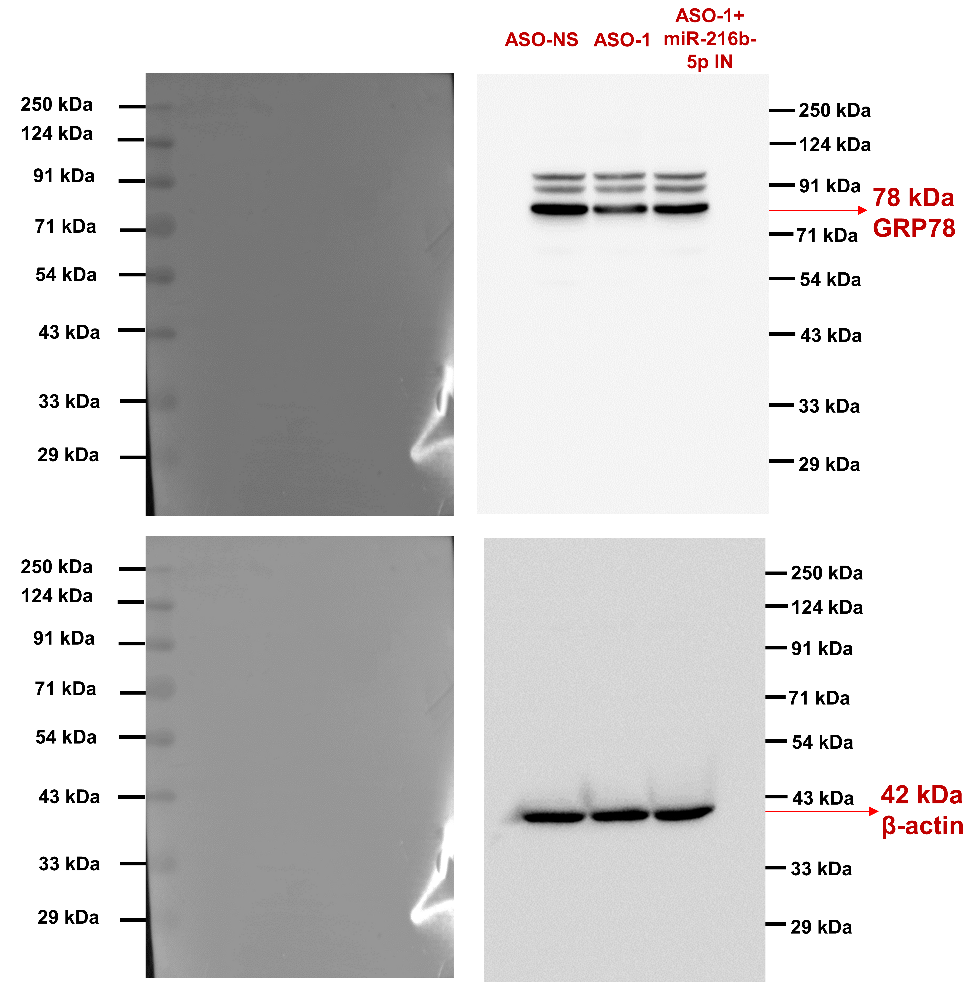
**

1. **Figure 6H– FaDu**

**
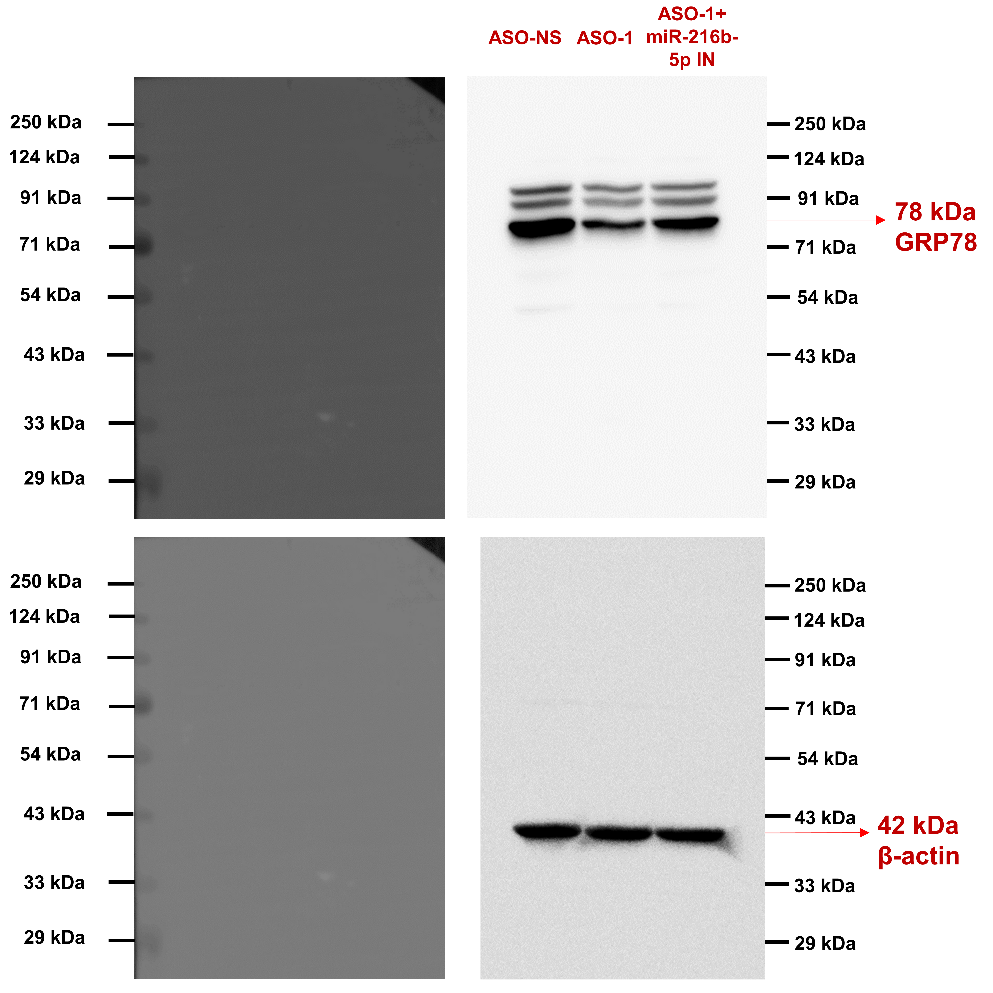
**

**References:**

1. Chandrashekar, D. S. *et al.* UALCAN: An update to the integrated cancer data analysis platform. *Neoplasia N. Y. N* **25**, 18–27 (2022).

2. Agarwal, V., Bell, G. W., Nam, J.-W. & Bartel, D. P. Predicting effective microRNA target sites in mammalian mRNAs. *eLife* **4**, e05005.

3. Sticht, C., Torre, C. D. L., Parveen, A. & Gretz, N. miRWalk: An online resource for prediction of microRNA binding sites. *PLOS ONE* **13**, e0206239 (2018).

4. Thul, P. J. & Lindskog, C. The human protein atlas: A spatial map of the human proteome. *Protein Sci. Publ. Protein Soc.* **27**, 233–244 (2018).

5. Shree, B., Tripathi, S. & Sharma, V. Transforming Growth Factor-Beta-Regulated LncRNA-MUF Promotes Invasion by Modulating the miR-34a Snail1 Axis in Glioblastoma Multiforme. *Front. Oncol.* **11**, (2022).

6. Shree, B., Sengar, S., Tripathi, S. & Sharma, V. LINC01711 promotes transforming growth factor-beta (TGF-β) induced invasion in glioblastoma multiforme (GBM) by acting as a competing endogenous RNA for miR-34a and promoting ZEB1 expression. *Neurosci. Lett.* **792**, 136937 (2023).
